# θ-γ Cross-Frequency Transcranial Alternating Current Stimulation over the Trough Impairs Cognitive Control

**DOI:** 10.1523/ENEURO.0126-20.2020

**Published:** 2020-09-04

**Authors:** Zsolt Turi, Matthias Mittner, Albert Lehr, Hannah Bürger, Andrea Antal, Walter Paulus

**Affiliations:** 1 Department of Clinical Neurophysiology, University Medical Center Göttingen, Göttingen 37073, Germany; 2 Department of Neuroanatomy, Institute of Anatomy and Cell Biology, Faculty of Medicine, University of Freiburg, Freiburg 79104, Germany; 3 Department of Psychology, UiT The Arctic University of Norway, 9037

**Keywords:** cognitive control, θ-γ cross-frequency coupling, transcranial alternating current stimulation

## Abstract

Cognitive control is a mental process, which underlies adaptive goal-directed decisions. Previous studies have linked cognitive control to electrophysiological fluctuations in the θ band and θ-γ cross-frequency coupling (CFC) arising from the cingulate and frontal cortices. However, to date, the behavioral consequences of different forms of θ-γ CFC remain elusive. Here, we studied the behavioral effects of the θ-γ CFC via transcranial alternating current stimulation (tACS) designed to stimulate the frontal and cingulate cortices in humans. Using a double-blind, randomized, repeated measures study design, 24 healthy participants were subjected to three active and one control CFC-tACS conditions. In the active conditions, 80-Hz γ tACS was coupled to 4-Hz θ tACS. Specifically, in two of the active conditions, short γ bursts were coupled to the delivered θ cycle to coincide with either its peaks or troughs. In the third active condition, the phase of a θ cycle modulated the amplitude of the γ oscillation. In the fourth, control protocol, 80-Hz tACS was continuously superimposed over the 4-Hz tACS, therefore lacking any phase specificity in the CFC. During the 20 min of stimulation, the participants performed a Go/NoGo monetary reward-based and punishment-based instrumental learning task. A Bayesian hierarchical logistic regression analysis revealed that relative to the control, the peak-coupled tACS had no effects on the behavioral performance, whereas the trough-coupled tACS and, to a lesser extent, amplitude-modulated tACS reduced performance in conflicting trials. Our results suggest that cognitive control depends on the phase specificity of the θ-γ CFC.

## Significance Statement

This study investigated the behavioral effects of different forms of θ-γ cross-frequency coupling (CFC) in cognitive control. To this aim, we delivered cross-frequency transcranial alternating current stimulation (tACS) over the cingulate and frontal cortices in humans. We found that when γ tACS was coupled to the trough of θ tACS, the stimulation worsened the ability of healthy participants to employ cognitive control. Our findings highlight the role of θ-γ CFC in complex goal-directed behavior in humans.

## Introduction

In goal-directed behavior, contextual and reward-related information should be effectively linked to form action plans to accomplish goals and perform decisions in a flexible and prospective manner (Helfrich and Knight, 2019). In humans, at least three main behavioral control systems influence the decisions: The Pavlovian system and the model-free and the model-based instrumental systems (Guitart-Masip et al., 2014). The Pavlovian system is responsible for automatic, reflexive response tendencies that depend on the valence of the stimulus. It facilitates approaching behavior for rewarding stimuli and response inhibition for unrewarding ones (Guitart-Masip et al., 2014). The model-free system gradually incorporates the behavioral consequences of actions by computing the difference between the predicted and received outcome. The model-based system creates an internal world model, which enables flexible, prospective planning. Therefore, decisions do not exclusively rely on the outcome history (Helfrich and Knight, 2019).

Conflict can arise between the Pavlovian and instrumental behavioral control systems, when the evolutionary hard-wired, valence-response associations do not support adaptive behavior. This situation occurs when approaching rewards is maladaptive, or when rewards can be secured by response inhibition rather than by approach (Guitart-Masip et al., 2012). Cognitive control is a mental process for resolving this conflict between the behavioral control systems (Guitart-Masip et al., 2014; Shenhav et al., 2017).

The oscillatory activity in the θ and γ frequency bands and their interaction may play a crucial role in cognitive control (Cavanagh and Frank, 2014; Cohen, 2014). θ-γ, phase-amplitude cross-frequency coupling (CFC) is one form of such interaction, where the phase of the θ oscillation modulates the amplitude of the γ oscillation (Canolty and Knight, 2010). Human intracranial electrophysiological recordings revealed that θ-γ, phase-amplitude CFC in the anterior cingulate cortex (ACC) and dorsolateral prefrontal cortex (DLPFC) emerges during cognitive control (Smith et al., 2015). Smith and colleagues found that the amplitude of the high γ oscillation was highest in a specific phase range of the θ oscillation (∼0–60°) during a cognitive control task (Smith et al., 2015).

To study how participants learn to overcome the Pavlovian bias by using cognitive control mechanisms, we used a probabilistic Go/NoGo instrumental learning task (Cavanagh et al., 2013). We tested the behavioral relevance of θ-γ CFC in humans via transcranial alternating current stimulation (tACS), which can externally generate oscillating electric fields in the brain (Peterchev et al., 2012). We used three CFC-tACS protocols delivered in the θ and γ frequency bands: peak-coupled and trough-coupled tACS and amplitude-modulated tACS (Alekseichuk et al., 2016; Minami and Amano, 2017; Amador de Lara et al., 2018). In the context of the present study, the notion of peak and trough refers to the local maximum and minimum of the amplitude of the delivered θ tACS wave, to which the short γ tACS burst was coupled. In the amplitude-modulated protocol, the amplitude of the γ oscillation was modulated by the phase of the θ wave.

We hypothesized that the peak-coupled tACS would improve the accuracy and/or the speed of learning relative to the control stimulation. We based this hypothesis on the notion that these protocols mimic the phase specificity of θ-γ CFC when signaling the need for cognitive control (Smith et al., 2015). Moreover, we also anticipated that the trough-coupled tACS would impair behavioral performance because this pattern is contrary to that activity naturally occurring during the successful implementation of cognitive control (Smith et al., 2015). Third, we expected that modulating the CFC between the ACC and DLPFC via CFC-tACS protocols should affect the amount of Pavlovian bias. In particular, facilitating the CFC between the ACC and DLPFC via the peak-coupled tACS would be thought to increase the efficacy of the ACC to signal the need for cognitive control and thereby increase the degree of model-based control implemented by the DLPFC (Smith et al., 2015). This, in turn, might lead to a decreased amount of Pavlovian bias. On the other hand, disrupting the CFC between the ACC and the DLPFC via the trough-coupled tACS should decrease the efficacy of signaling the need for cognitive control. This may impair the efficacy of implementing model-based control and therefore lead to a higher degree of Pavlovian bias. Fourth, we expected that amplitude-modulated tACS would improve behavioral performance by entraining the ongoing θ oscillation by the envelope of the high-frequency stimulation (Negahbani et al., 2018). The amplitude-modulated tACS protocol would increase the θ synchrony in the cingulate and frontal cortices (Negahbani et al., 2018), which in turn would improve the ability of the participants to apply cognitive control.

## Materials and Methods

### Participants

Twenty-four healthy, native German-speaking adult volunteers (12 female, mean age ± SD: 23.0 ± 3.26 years, age range from 18 to 30 years) joined the study. This number of participants was chosen to allow a complete randomization of the order of the four tACS protocols (i.e., three active and one control protocols) and is calculated as four factorial or 24. The mean number of years of education (±SD) was 16.30 ± 3.05 (range from 12 to 22.5 years). Before entering the study, the participants were informed about possible adverse effects of tACS, and all of them gave their written informed consent. The exclusion criteria were history or presence of current medical, neurologic, or psychiatric illnesses, including epilepsy, drug and/or alcohol addiction, and the presence of metal implants in the head, neck, and chest. In addition, the participants were examined by neurologists at the Department of Clinical Neurophysiology, University Medical Center Göttingen. The study neurologist evaluated whether any of the exclusion criteria were met. None of the participants reported any neurologic or psychiatric disorders, drug dependency, or medication acting on the central nervous system before or during the experiment.

### Code accessibility, data availability, and ethic statement

The Ethics Committee of the University Medical Center Göttingen approved the study, the study protocols, and all methods used therein. We performed the study in accordance with relevant guidelines and regulations. The study was registered under the study approval number 20/5/15. The study materials, code/software and pseudonymized raw data described in the paper is freely available online at https://github.com/ihrke/2020_cfc_tacs.

### Experimental design

The study used a double-blind, within-subject design. The participants underwent five experimental sessions, starting with an initial training session to familiarize themselves with the behavioral paradigm. During the training session, the participants received no stimulation. This initial session was followed by the four tACS sessions, the order of which was counterbalanced across participants to reduce between-session learning effects. Of the four stimulation sessions, three employed the main stimulation protocols and one the control protocol. The intersession interval between the stimulation sessions was at least 48 h.

### Behavioral paradigm

**Figure 1.**
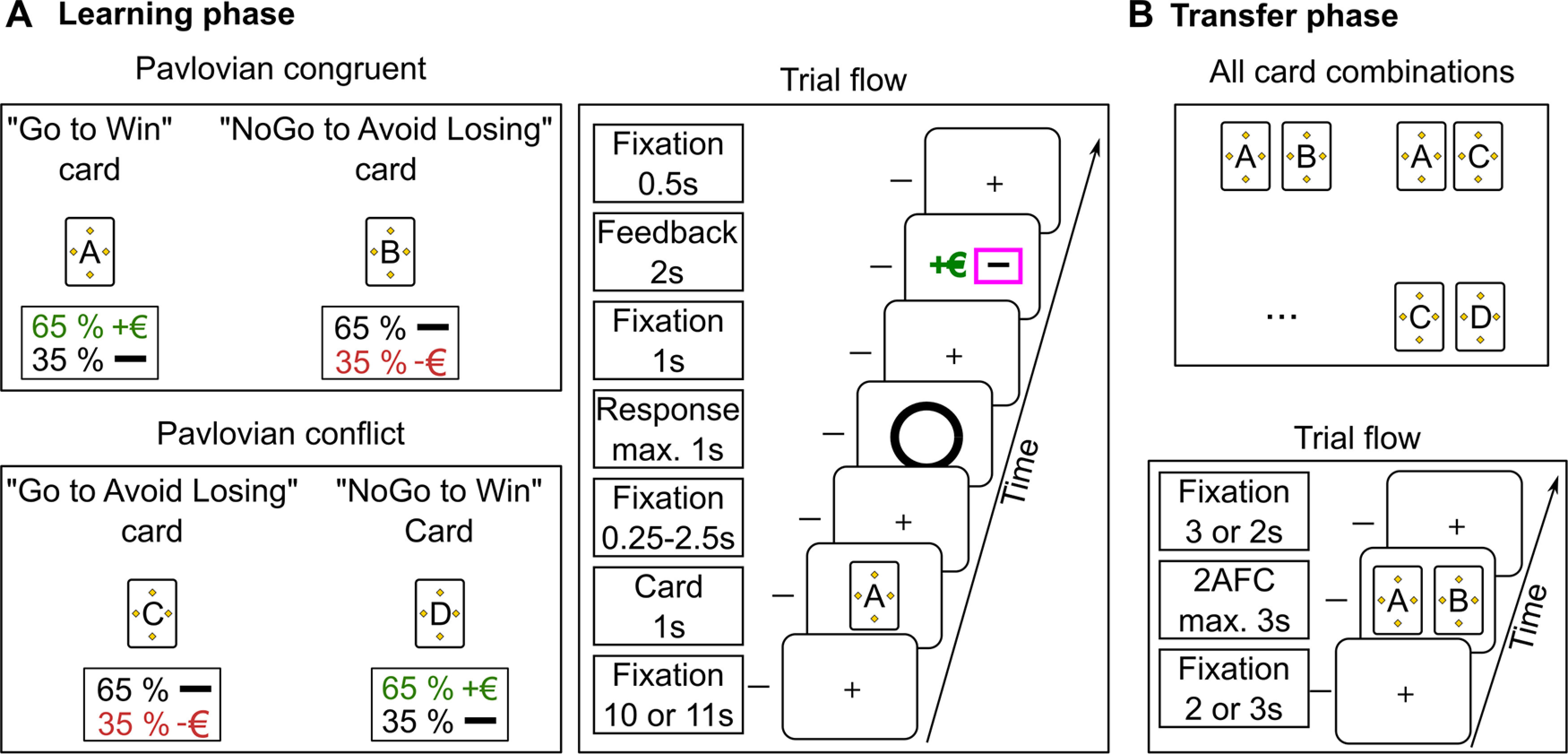
The structure and the trial flow of the behavioral paradigm for the learning (***A***) and the transfer phase (***B***).

The behavioral paradigm consisted of a learning phase and a subsequent transfer phase, which was adapted from Cavanagh et al. (2013). The task was introduced as a card game for the participants (Fig. 1). Stimuli presentation was controlled by PsychoPy (version number 1.83.01), a free, open-source application built on the Python programming language (Peirce, 2007, 2009). For the presentation of the behavioral paradigm, we used a Dell computer with Windows 7 Enterprise 64-bit operating system, Intel (R) core i3-3220, 3.30 GHz and 4 GB RAM, and a 21.5-inch Dell screen with a 1920 × 1080 resolution and 60-Hz refresh rate.

During the learning phase, the participants performed a Go/NoGo instrumental learning task. Here, they had to learn action (two levels: Go/NoGo) and monetary outcome (three levels: win, no win/lose, or lose) contingencies. For each card, the goal was to find the better of the two possible action choices (Go/NoGo) resulting in the highest monetary outcome (getting reward or avoiding losing) and therefore maximize their earnings.

One key feature of the task was that the action choices and monetary outcomes were orthogonal. As such, the four unique cards covered all the combinations between actions choices and monetary outcomes (“Go to win,” “NoGo to avoid losing,” “Go to avoid losing,” and “NoGo to win”). Because of the Pavlovian bias, i.e., approach to appetitive and withdrawal from aversive stimuli, the cards could be split into congruent and conflicting cards. For the Pavlovian congruent cards (henceforth the congruent cards; “Go to win” and “NoGo to avoid losing”), the action selection under the automatic, Pavlovian bias was advantageous for the participants and hence easier to learn. For the Pavlovian conflicting cards (henceforth the conflicting cards; “NoGo to win” and “Go to avoid losing”), the action selection under the automatic, Pavlovian bias was disadvantageous for the participants and therefore harder to learn (Guitart-Masip et al., 2012).

The action outcomes were probabilistic such that 65% of correct responses led to a better outcome: neutral monetary outcomes (no loss) for the lose cards and monetary reward for the win cards. Consequently, 35% of the correct responses led to neutral monetary outcomes for the win cards and monetary loss for the lose cards. On the other hand, wrong responses inverted this ratio, i.e., 65% of incorrect responses led to neutral monetary outcomes for the win cards and monetary loss for the lose cards. Previous studies used 80% versus 20% or 70% versus 30% action-outcome contingencies, which renders the present version of the probabilistic learning task slightly more difficult compared with previous versions (Cavanagh et al., 2013; Guitart-Masip et al., 2012; Csifcsák et al., 2020).

For illustrative purposes, we describe possible action-outcome scenarios. Suppose card A was a “Go to win” card, a fact unknown to the participant. In case the participant decided to take the card, there was a 0.65 probability to receive the feedback indicating monetary reward. Consequently, there was a 0.35 probability to receive no reward. In case the participant did not take the “Go to win” card, the feedback probabilities were reversed. That is, the probability of receiving monetary reward was 0.35 and the probability of receiving no reward was 0.65.

Each card was presented 20 times in a random order. Independent sets of five cards were used and randomly chosen for each session from a pool of six sets of cards. We created six card sets for the scenario that one session has to be repeated. Therefore, participants performed 80 trials in each session (20 trials × four cards) and 400 trials in total (80 trials × five sessions).

The presentation of the stimuli was performed in full screen mode. We set the background color of the screen to white. At the beginning of each trial, a black fixation cross (10 or 11 s) was presented (Fig. 1*A*, trial flow). Note that we used a relatively long duration of fixation cross in the present study compared with previous studies (Guitart-Masip et al., 2012). Also, during this time the participants were instructed to blink and swallow. This was a necessary step to increase the comparability of the present results with our other experiments using pre-stimulus intermittent tACS (manuscript in preparation) and scalp electroencephalogram recordings.

Then a card cue (1 s; original image size 199 × 279 pixels, presentation size 0.3 × 0.5) was presented to the participants. We used white cards and distinguished them with a black capital letter (B, C, D, F, G, H, J, K, R, S, T, V, A, E, O, U, L, M, P, Q, W, X, Y, Z) printed in the middle of the card (Fig. 1*A*, trial flow). We decorated the cards by adding four pieces of simple shapes around the letter. We used rhombus, circle, and rectangle shapes and filled them with blue, gray, green, pink, orange, or yellow colors. In each set, we used the same shape and color for each card.

The target detection stimulus (black circle; original image size 225 × 220 pixels, presentation size 0.35 × 0.45) was shown until a response occurred, or 1 s passed. The target detection stimulus indicated to the participants that they could take the card (Go) or not (NoGo), on which the monetary outcome depended. The feedback was displayed (original image size 402 × 205 pixels, presentation size 0.6 × 0.4) for 2 s: a green “+€” sign indicated a monetary reward, a red “–€” symbol indicated a monetary loss and a black horizontal bar indicated neutral monetary outcome (neither win nor loss). The next trial started 0.5 s after feedback.

In the subsequent transfer phase of the task (Fig. 1*B*), the participants performed a two-alternative, forced-choice (2AFC) task where each card from the learning phase was paired with one of the three other cards following the order (e.g., “Go to win” vs “NoGo to avoid losing,” “NoGo to avoid losing” vs “Go to win,” etc.). Each of the 12 card pairs was presented four times until a response occurred, or 3 s passed.

The dependent variable in this study was accuracy. We defined accuracy as choosing the response category (Go/NoGo) that led with a higher probability to the better monetary outcome; hence, monetary reward for the win cards and neutral monetary outcome for the losing cards.

The participants were paid eight Euros/hour and received an additional performance-dependent bonus of 12 Euros if their mean performance calculated over all sessions was above 75%. We used the monetary bonus to encourage our participants to perform as well as possible in each session. Unknown to the participants, everybody received the monetary bonus at the end of the experiment.

### tACS

**Figure 2.**
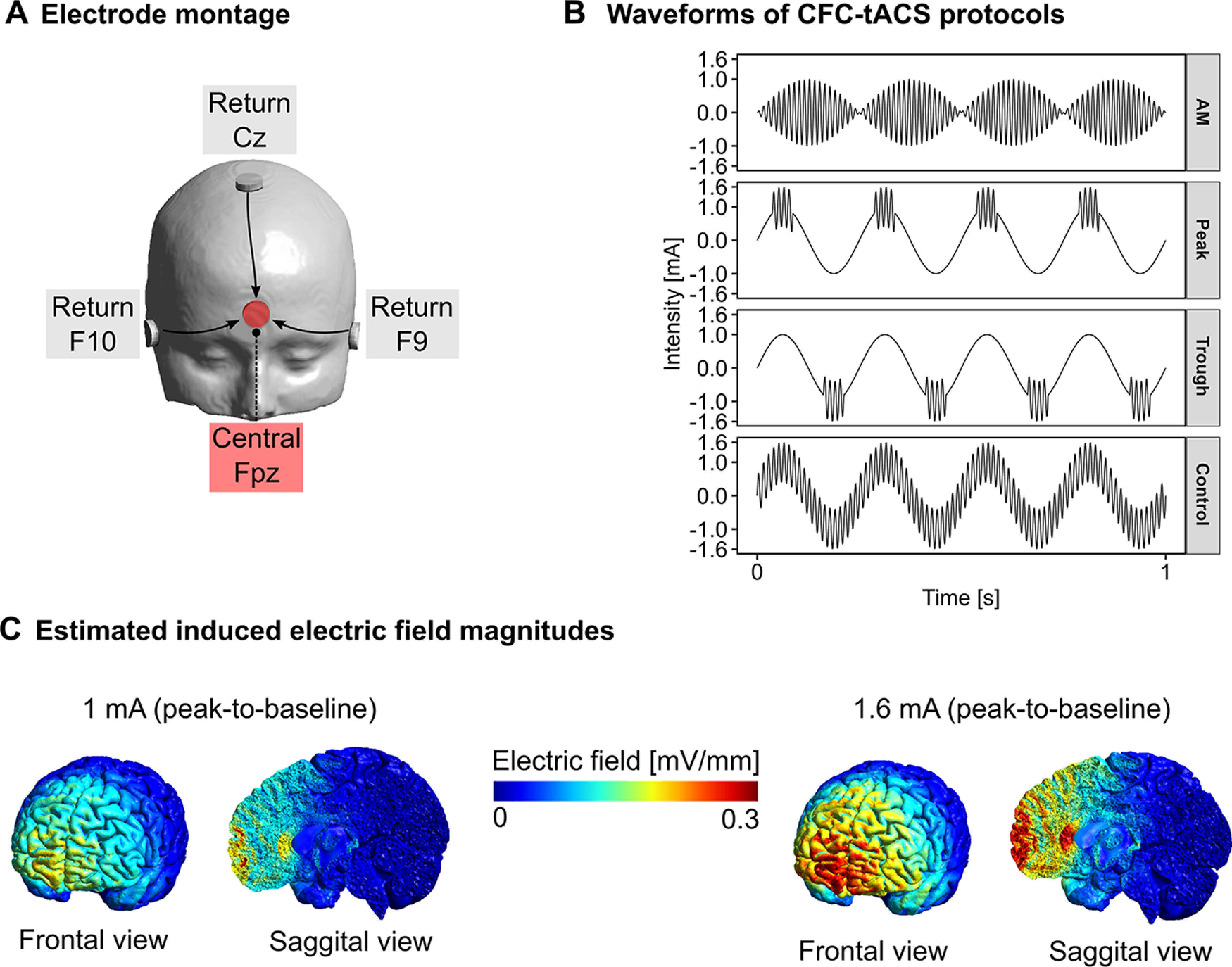
Stimulation parameters including electrode montage (***A***), cross-frequency-coupling tACS waveforms (***B***), and estimated electric field magnitudes in the gray matter (***C***). Electric field simulations were performed with SimNIBS version 3.0.2 on a template head model. The peak absolute electric field strength reached 0.3 ^mV^/_mm_ in the medial frontal cortex. AM, amplitude modulated; Peak, peak-coupled tACS; Trough, trough-coupled tACS; Control, control tACS.

The stimulation was delivered by a CE-certified NeuroConn multichannel stimulator (neuroConn GmbH) during the learning phase of the task. The electrode positions were chosen according to the international 10–20 EEG system. The electrode montage was centered over the Fpz electrode location with three return electrodes positioned over the Cz, F10, and F9 positions (Fig. 2*A*).

The following standardized steps ensured minimal stimulation-induced cutaneous sensations. After determining the electrode locations, the corresponding skin surface was gently cleaned with OneStep abrasive gel (H + H Medizinprodukte GbR), which was removed with 0.9% saline solution (B. Braun Melsungen AG). After removing the residual saline solution with paper tissue, a local anesthetic cream (Anesderm, Pierre Fabre Dermo-Kosmetic GmbH) was applied for 20 min to numb the skin (25 mg/g lidocaine, 25 mg/g prilocaine). It was wiped off first with paper tissue followed by a skin antiseptic spray (Kodan Tinktur Forte, Schuelke & Mayr GmbH). The latter was necessary to remove the anesthetic cream, which would otherwise prevent the conductive paste from adhering to the skin. Homogenous layers of Ten20 conductive paste (Waever and Company) were then applied to the skin and the electrode surfaces. Each of the four round, conductive rubber electrodes with 2-cm diameter (neuroConn GmbH) was affixed to the head. The impedance was kept below 10 kΩ. The maximal current density under the main electrode was 0.50 mA/cm^2^. The electrode montage was prepared in a double-blind fashion.

We used four different CFC-tACS protocols, including amplitude-modulated CFC-tACS (AM), CFC over the peak, trough, and control tACS (Fig. 2*B*). Each protocol started with a 20-s fade-in period, followed by a 20-min stimulation with the maximum stimulation intensity, and ended with a 10-s fade-out period. The total stimulation duration was 20 min and 30 s.

The protocols, peak-coupled, trough-coupled tACS and control, consisted of a 4 Hz, 1 mA (=2 mA peak to peak) sinusoidal waveform coupled with a 0.6 mA (=1.2 mA peak to peak) 80-Hz sinusoidal waveform. These stimulation protocols had a maximum intensity of 1.6 mA. In the peak-coupled tACS protocol, the short 80-Hz burst (50 ms) was coupled over the peak (38–88 ms) of each θ cycle. In the trough-coupled tACS protocol, the short 80-Hz burst was coupled over the trough (163–213 ms) of each θ tACS cycle. In the control stimulation, both waveforms were overlaid continuously. The control stimulation lacked any phase specificity of γ relative to θ oscillations but used a highly matched intensity range and identical stimulation duration with respect to the real protocols. The control protocol served as the reference to which we compared the effects of the three main CFC-tACS protocols.

In the AM protocol, the amplitude of the γ frequency (80 Hz) was modulated by the phase of the θ frequency (4 Hz). In all protocols, the amplitude of the θ frequency was constant. Consequently, the AM protocol employed lower peak stimulation intensities (Fig. 2*C*, left) compared with the remaining protocols, which led to a slightly higher electric field strength (Fig. 2*C*, right). However, this was a necessary step to match the amplitude of the envelope frequency in the amplitude modulation protocol to the amplitude of the θ frequency in the remaining protocols.

In order to estimate the magnitude of the induced electric field in the brain, we ran simulations using the free software package Simulations for Non-invasive Brain Stimulation (SimNIBS; version 3.0.2; Thielscher et al., 2015). To this aim, we conducted electric field calculations on an anatomically realistic, six-compartment template head model (almi5.msh) available in SimNIBS. We used default conductivity values [S/m] that were set to 0.465 for the scalp, 0.01 for skull, 1.654 for cerebrospinal fluid, 0.275 for gray matter, and 0.126 for the white matter. The simulation accounted for volume-normalized anisotropy in the brain. We observed peak electric field magnitudes up to 0.3 ^mV^/_mm_ in the medial frontal cortex (Fig. 2*C*, right).

### Procedure

At the start of each session, the participants filled out a short questionnaire. We asked our participants to report the quality of sleep during the previous night. Further, we assessed the level of arousal (“How are you feeling right now?”) with a 10-point Likert-scale where value 1 corresponded to very tired and 10 to completely awake. We also assessed the presence and intensity of headache (“Do you have a headache right now?”) with an initial yes-no answer and an optional 10-point Likert-scale for yes responses. Here, value 1 corresponded to low and 10 to very strong headache. We assessed the intake of medication, coffee, or alcohol consumption in the 24 h before the session. The purpose of these assessments was to avoid the possibility that irregular sleep patterns in the previous night, headache or mental fatigue because of alcohol would corrupt the possible behavioral findings of tACS. Theoretically, a new session was going to be scheduled if the participant had consumed more than two alcoholic beverages in the previous day, however, arranging a new session was not necessary.

All participants received detailed written instructions about the task. Before the training session, we asked them to perform a practice session to familiarize themselves with the task and to ensure that they were able to operate the response box (RB-740, Cedrus Corporation) comfortably. We used an independent set of cards in the practice session. Before the start of the learning task, the participants filled out a questionnaire to ensure that they understood the tasks correctly. The questionnaire assessed whether the participants understood (1) the meaning of the three feedback types (win, no win/no loss, loss) and (2) the probabilistic nature of the feedback.

In the following stimulation sessions, the short questionnaire was followed by the electrode preparation, the application of the topical anesthetic cream, and the impedance measurements. This preparation phase took ∼35–40 min, during which the participants watched documentary movies to maintain their vigilance.

Following the preparatory phase, the participants performed two short practice tasks. The practice tasks contained 16 trials for the learning and 12 trials for the transfer phase.

Following the practice task and directly before the start of the learning task, the data collector opened the sealed envelope containing the information about that day’s stimulation condition. After opening the envelope, the data collector selected the protocol on the stimulator and informed the participants about the start of the stimulation. Following this moment, the data collector initiated no further communication. The learning phase began directly after the fade-in period. After the end of learning phase and following a 5-min break, the participants completed the transfer phase of the task, during which no stimulation was applied.

At the end of each session, we assessed the level of self-reported arousal, the presence and intensity of headache and secondary perceptual adverse effects associated with the application of tACS. We focused on cutaneous (i.e., itching, tingling, and burning) and visual flickering sensations (i.e., phosphenes). First, the participants were asked to indicate the presence of secondary adverse effects (yes or no question). In case of a positive answer, we assessed the subjective level of discomfort using a 10-point Likert scale. On the Likert scale, “1” indicated the lowest noticeable discomfort, and “10” indicated an amount of discomfort the participants would not be able to endure during the experiment. The participants were informed that they could discontinue the study at any time without having to give any reason for terminating the study.

At the end of each session, we asked our participants to recall the card types and provide an internal ranking of the cards. We focused on whether the participants were able to correctly recall the cards’ valence-action contingency.

### Statistical analysis

All statistical analyses were performed using the R statistical programming environment (version 3.5.1) and the RStudio integrated development environment (version 1.1.456; R Studio Team, 2016; R Core Team, 2018). For the data analysis, we used a Precision 7920 Rack computer, Debian GNU/Linux 9.9 operating system, 2 × Intel Gold 6152, 2.1 GHz, 22 cores, and 512 GB RAM.

We applied Bayesian methods, and we report our results in terms of the mean of the posterior distribution and their associated 95% highest-density intervals (HDIs). These intervals are derived from the posterior distribution of the model-parameters or a combination of parameters (e.g., differences) by finding the interval that contains 95% of the posterior mass while also satisfying the criterion that all points within the interval have a higher probability density than points outside the interval (Kruschke, 2014). The interpretation of the Bayesian 95% HDI is that it gives the range in which the estimated parameter is located with a probability of 0.95. We consider effects to be statistically reliable, if the 95% HDI excludes zero.

In order to model accuracy on the single-trial level, a dichotomous dependent variable, we used hierarchical Bayesian logistic regression. For these regression analyses, we used the R package *brms* (Bayesian Regression Models using Stan; Bürkner, 2018) with default, uniform priors for all regression coefficients. This package uses Hamiltonian Monte-Carlo (HMC) techniques implemented in Stan (Carpenter et al., 2017) to fit the models. We used four chains, where each chain had a warm-up period of 1000 samples and 1000 post warm-up samples resulting in a total of 4000 posterior samples. We used the Gelman–Rubin diagnostic (Gelman and Rubin, 1992) to ensure that all reported results had an $\hat{R}\leq 1.05$. For model comparison, we used the Leave-One-Out Information Criterion (LOOIC), where lower scores of the LOOIC suggest a better model fit (Vehtari et al., 2017). Specifically, a model was considered better if the LOOIC score were lower, and if the ΔLOOIC score were at least double the corresponding LOOIC SE.

### Computational modeling

The orthogonal Go-NoGo task used in our study usually allows one to fit computational reinforcement learning (RL) models to the data collected during the experiment (Cavanagh and Frank, 2014; Csifcsák et al., 2020). These models assume that each time a certain stimulus is encountered, an internal value representation of the stimulus-action pair (known as *Q* value) is updated according to the reward received after taking an (in-)action. Furthermore, the decision on which action to take is based on this internal value-representation, and thus, as the *Q* value gets close to the actual value with repeated encounters of a stimulus, performance becomes more accurate. The orthogonalized nature of the Go-NoGo task typically also allows the estimation of Pavlovian influences on this RL process by biasing Go responses for rewarding stimuli and NoGo responses for punished stimuli. We used Bayesian hierarchical modeling to fit a series of these models to our data using a strategy identical to that presented in Csifcsák et al. (2020), and we refer the reader to this paper and the data repository for this paper at https://github.com/ihrke/2020_cfc_tacs for technical details of the RL model. The model-code was based on a the hBayesDM toolbox (Ahn et al., 2017).

The described computational models were implemented using the R-package rstan (Stan Development Team, 2018). We used eight parallel chains with a total of 8000 postwarm up samples from the posterior distribution. The convergence diagnostics were identical to the other models as described above.

## Results

### Computational modeling

We fitted models of increasing complexity to the data from our experiment. First, we fitted a model without any session-specific terms (null-model) as a baseline. Next, we modeled separate learning-rates α, temperature parameters β, Pavlovian bias parameters π and go-biases b for each of the tACS sessions (tACS-model). Furthermore, we included a model that let each of the four core-parameters depend on the session order (order-model) and, finally, a model where separate parameters were fit for each tACS session and each parameter depended on session-order (full model). Diagnostics of the HMC chains indicated that all models converged successfully.

We calculated the LOOIC for each of these models (Table 1). Although the model that only modeled the RL parameters as a function of session order received the lowest LOOIC, the differences between all four models were small compared with their SEs (Table 1) and model selection was therefore inconclusive. We conducted posterior predictive checks and simulated 1000 random datasets from the posterior distribution of the parameters. Unfortunately, while some general characteristics of our participants’ performance were captured by the model, it failed to properly account for the complex changes across sessions, trials, and card types. Given that the computational models were unable to capture our participants’ behavior, we chose not to interpret or report changes in model parameters across sessions but to focus on the more descriptive logistic regression models reported below. The reason for our failure to model our participants’ performance with these established models is puzzling and deserves further investigation.

**Table 1:** Results of the model selection procedure for the computational models

Model	LOOIC	ΔLOOIC	SE(ΔLOOIC)
Order	10,598.3	–	–
Full	10,607.8	9.6	30.2
tACS	10,608.5	10.2	39.4
Null	10,615.3	17.0	33.2

All differences in LOOIC are small compared with their SEs and model selection is therefore inconclusive.

### Accuracy and learning

To assess learning performance across sessions, we fitted a series of hierarchical Bayesian logistic regression models, treating accuracy as the dependent variable. All of the models received a random intercept for each participant and for sessions nested within participants. Furthermore, we included various combinations of the following predictor variables: Card type (four levels: Go-to-Win, NoGo-to-Avoid, Go-to-Avoid, and NoGo-to-Avoid), tACS session (five levels: Training, Control, AM, Peak, and Trough), Trial (Z-transformed trial number during each experimental session), session order (continuous predictor coding for the order in which the tACS sessions were conducted) as well as their interactions. All of these 20 models were compared according to their out-of-sample predictive performance using the LOOIC (Vehtari et al., 2017). Based on this criterion, we calculated model weights using two different techniques: based on Akaike weights (Wagenmakers and Farrell, 2004) using the LOOIC instead of the AIC and using Bayesian model averaging (BMA; Yao et al., 2018). Both of these techniques resulted in posterior probabilities quantifying how likely it is that each of the models was the best one.

After calculating these model selection criteria, we found converging evidence that the model that encompassed all predictors, including all two-way and three-way interactions between Card, tACS session and Trial, as well as a main effect of Session order outperformed the other models (Akaike weight p=0.63, next best model p=0.34; BMA weight p=0.47, next best model p=0.23).

**Figure 3.**
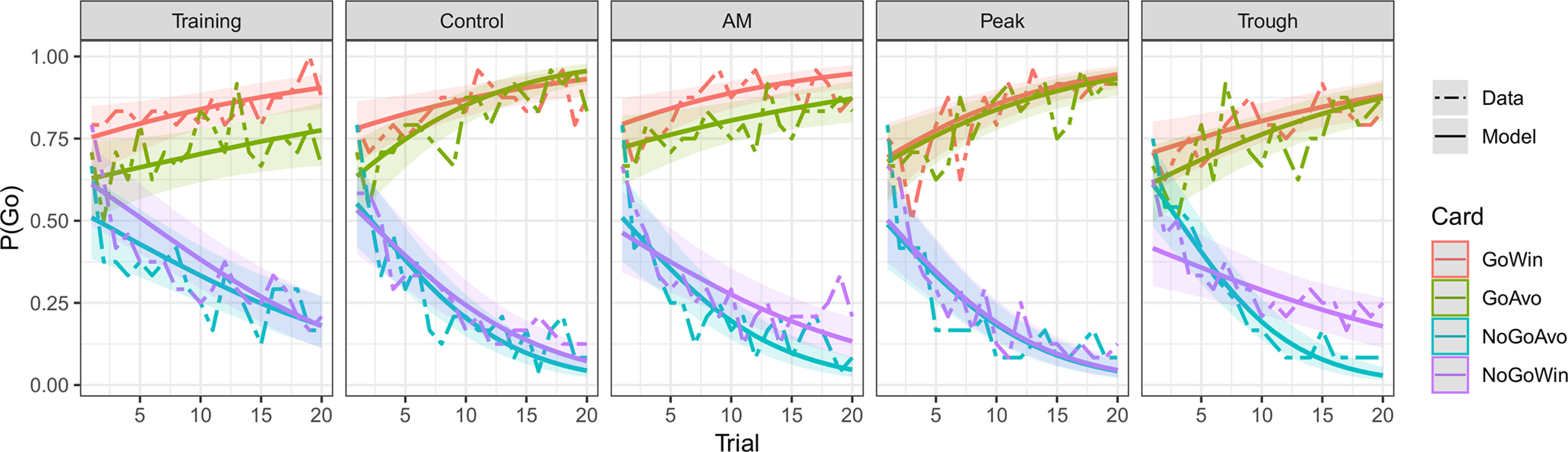
Posterior predictive checks for the final logistic regression model. The model predictions (solid lines) captured the main trends in the data (dashed lines) well. Colored ribbons are 95% HDIs. AM, amplitude modulated; Control, control tACS; Peak, peak-coupled tACS; Trough, trough-coupled tACS. GoWin: Go-to-Win, GoAvo: Go-to-Avoid, NoGoAvo: NoGo-to-Avoid, NoGoWin: NoGo-to-Win.

We therefore based our conclusions on that winning model and investigated it in detail. First, we checked that the model captured the trends in the data well. In Figure 3, we plotted the raw data and overlaid predictions from the winning logistic regression model (posterior predictive check). The model captured the trends in the data well and the uncertainty (95% HDIs) around the model-predictions was sufficiently broad relative to the fluctuations in the data. The Bayesian $R^{2}$ value for this model was $R^{2}=0.23$ HDI [0.22,0.24].

We focused on two separate aspects of the data. First, we investigated how the general accuracy level varied across cards and sessions. In the presence of the three-way interaction of Card × tACS session × Trial, we quantified and compared the accuracy level in the middle of each session. Second, we were interested in the learning rate with which accurate responding increased. In our model, this was manifested in the tACS session × Trial, Card × Trial, and Card × tACS session × Trial interactions that allowed us to investigate the rate with which the correct way to respond to each of the cards was learned across the sessions.

### Average accuracy

**Figure 4.**
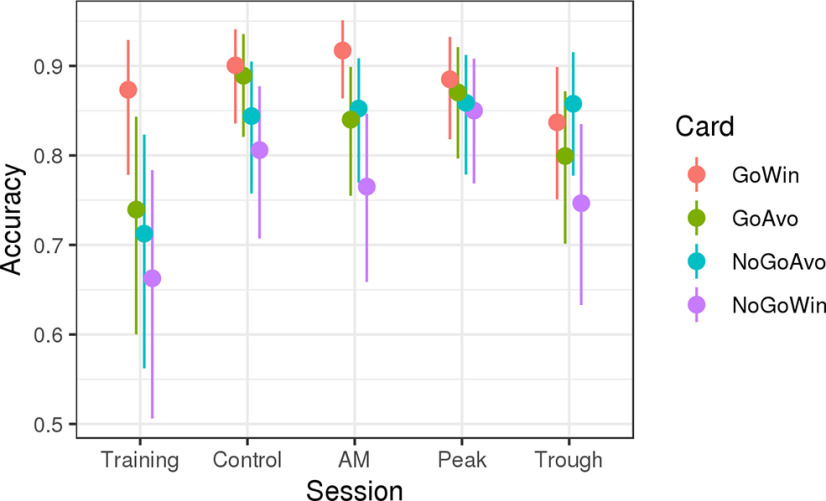
Estimated accuracy levels in the middle of the experimental session for each session and card. The colors represent the four card types, with the experimental sessions shown on the horizontal axis. Note that the participants received no tACS during the training session. AM, amplitude-modulated tACS; control, control tACS; Peak, peak-coupled tACS; Trough: trough-coupled tACS. GoWin: Go-to-Win, GoAvo: Go-to-Avoid, NoGoAvo: NoGo-to-Avoid, NoGoWin: NoGo-to-Win.

The accuracy levels as estimated by the model in the middle of each session are displayed in Figure 4. There was a significant amount of variation both between the cards and sessions. As expected, responses to the Go-to-Win card were generally most accurate ($b_{GoAvo}=-0.88\left[-1.24,-0.54\right]$, $b_{NoGoAvo}=-1.02\left[-1.39,-0.68\right]$, $b_{NoGoWin}=-1.25\left[-1.63,-0.90\right]$), while the NoGo-to-Win card was most difficult with the other two cards being situated between.

Furthermore, we found a learning effect between the Training session (which was always the first session each participant was exposed to) and the other sessions (which were randomized): performance was better in all tACS sessions and for all cards, the only exception being the Go-to-Win card in the Trough session (P(Trough>Training∨GoWin)=0.23). This learning-effect was not surprising given that this task is known to exhibit between-session learning effects (Csifcsák et al., 2020). However, after the initial effect of learning from the Training session to the second one, there was no clear further effect of Session order, $b_{order}=-0.08\left[-0.26,0.10\right]$.

**Figure 5.**
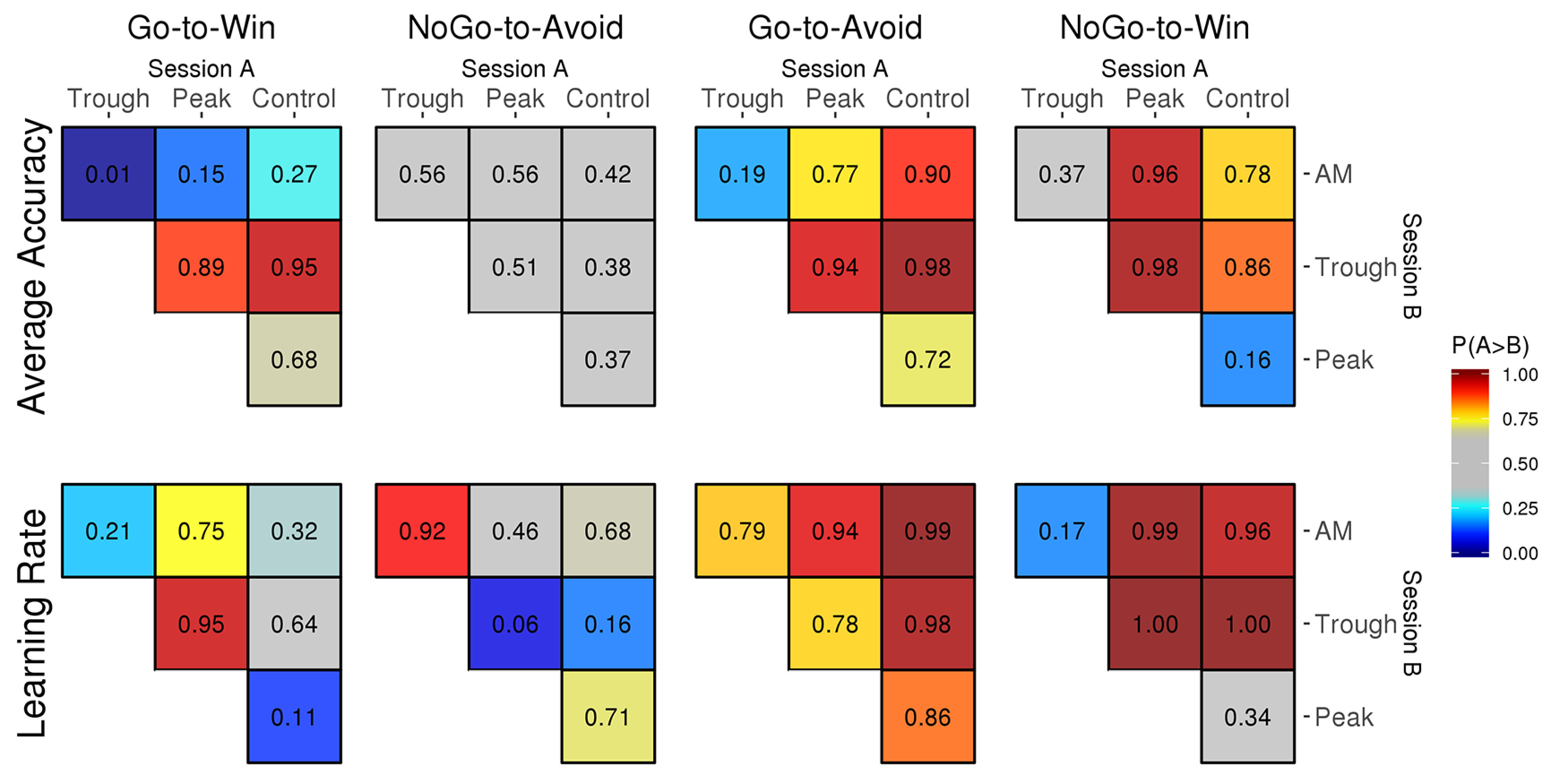
Comparison of average accuracy (top row) and learning rate (bottom row) between tACS sessions for each of the four cards. Colors and numbers in the matrices indicate the probability that the session indicated by the column showed a stronger effect compared with the session indicated by the row of each matrix. AM, amplitude modulated; Trough, trough-coupled tACS; Peak, peak-coupled tACS; Control, control tACS.

We were interested in how general accuracy changed between the different tACS sessions. A summary of the results is presented in Figure 5, upper row. Here, each entry in the matrix documents the posterior probability that accuracy was increased from one session (A) to the next (B). High values close to 1 (red) indicate that session A was highly likely to show increased accuracy relative to session B, while low values close to zero indicate the opposite. Intermediate values (gray) mean that the results are inconclusive for that particular comparison. For example, in the “Go to win” card, the value of 0.95 in the middle row, right column suggests that it is highly probable that the average accuracy was higher in the control tACS (session A) compared with the trough-coupled tACS (session B).

We start by comparing the three active tACS sessions AM, Peak, and Trough with the Control session. The *p* values given here represent the posterior probability that the active session showed higher accuracy compared with the Control session (i.e., the probability that the difference b is positive) and are not to be confused with frequentist *p* values.

There was no clear difference between the AM and the Control session for congruent cards (Go-to-Win: b=0.21[−0.47,0.87],p=0.73, NoGo-to-Avoid: b=0.06[−0.54,0.75],p=0.58) with possibly a small performance decrease for conflicting cards (Go-to-Avoid: b=−0.43[−1.09,0.19],p=0.10, b=−0.25[−0.85,0.38],p=0.22), although the HDIs for these effects did not exclude zero. The Peak session did not result in a change in general accuracy compared with Control for congruent (Go-to-Win: b=−0.15[−0.86,0.47],p=0.32, NoGo-to-Avoid: b=0.11[−0.52,0.76],p=0.63) or conflicting cards (Go-to-Avoid: b=−0.19[−0.84,0.47],p=0.28, NoGo-to-Win: b=0.31[−0.32,0.95],p=0.84). Finally, the Trough session showed reduced accuracy particularly for the easiest Go-to-Win cards, b=−0.56[−1.18,0.11],p=0.05 (but not for NoGo-to-Avoid, b=0.11[−0.55,0.74],p=0.62) and reduced accuracy for both conflicting cards (Go-to-Avoid: b=−0.70[−1.33,−0.04],p=0.02, NoGo-to-Win: b=−0.34[−0.95,0.29],p=0.14). Direct comparisons between the active stimulation sessions are also shown in Figure 5, upper row.

### Learning rate analysis

Next, we supplemented the analysis of the general accuracy with a parallel analysis regarding the learning rate, i.e., Card and tACS session interactions with the Trial term in the model. In Figure 5, the lower row shows a summary of this analysis. AM and Control sessions did not differ clearly for congruent cards (Go-to-Win: b=0.10[−0.30,0.52],p=0.68, NoGo-to-Avoid: b=−0.09[−0.47,0.31],p=0.32), but learning was decreased for conflicting cards (Go-to-Avoid: b=−0.50[−0.88,−0.13],p=0.01, NoGo-to-Win: b=−0.33[−0.69,0.02],p=0.04). For the Peak session, the results are similar but less clear, with a possible small improvement for Go-to-Win cards (b=0.24[−0.14,0.58],p=0.89) but not NoGo-to-Avoid (b=−0.11[−0.51,0.28],p=0.29) and possibly a weak decrease for Go-to-Avoid cards (b=−0.21[−0.61,0.18],p=0.14) but not for the NoGo-to-Win cards (b=0.08[−0.30,0.44],p=0.66). For the Trough session, we found no clear differences for congruent cards (Go-to-Win: b=−0.07[−0.41,0.31],p=0.36, NoGo-to-Avoid: b=0.21[−0.22,0.59],p=0.84) but clear learning decreases for the conflicting cards (Go-to-Avoid: b=0.36[−0.01,0.74],p=0.02, NoGo-to-Win: b=0.49[0.11,0.80],p=0.00).

### Perceptual adverse effects

Most participants reported no cutaneous sensations during tACS, possibly because of the application of the topical anesthetic cream. However, we also inspected the amount of perceptual adverse effects, such as itching, tingling, and burning sensations, and phosphenes that were reported following each tACS session. A careful inspection of the subjectively reported perceptual adverse effects did not reveal any substantial differences between the stimulation sessions.

## Discussion

In this study, we investigated the behavioral effects of three active θ-γ CFC-tACS protocols in a cognitive control task. In the peak-coupled and trough-coupled tACS conditions, we coupled the short bursts of 80-Hz γ tACS to the local maximum, i.e., peak, or minimum, i.e., trough, of the 4-Hz θ tACS. In the amplitude-modulated tACS condition, we modulated the amplitude of the 80-Hz γ tACS by the phase of the 4-Hz θ tACS. In the fourth condition, which served as a control, we continuously coupled the 80-Hz γ tACS to the 4-Hz θ tACS.

As we had hypothesized, we found that the trough-coupled tACS condition impaired behavioral performance, in particular in the more challenging, conflicting trials. We speculate that this protocol likely interfered with the phase-dependent θ-γ coupling between the cingulate (e.g., ACC) and the prefrontal cortices (e.g., DLPFC; Smith et al., 2015). In a previous study using a Stroop-like interference task, information transfer analysis (Granger causality) showed that the feedback-related information travels from the ACC to the DLPFC in the θ band (Smith et al., 2015). These findings may suggest that the ACC presumably signals the need for cognitive control, whereas the DLPFC processes this information and influences ongoing behavior by exerting model-based behavioral control (Smith et al., 2015). Thus, the modulation of the information flow from the cingulate to prefrontal cortex via θ-γ CFC could have impaired the model-based control in the trough-coupled tACS condition.

The observed behavioral effects in the present study may be because of the direct stimulation of the frontal and cingulate cortices or to indirect network effects. It has been shown in primates that there are monosynaptic connections between the frontal cortex, including the ventromedial prefrontal and cingulate cortices, to the subthalamic nucleus (Haynes and Haber, 2013). This pathway is called the hyperdirect pathway, which supposedly exerts a strong top-down control on ongoing decisions: it influences whether an action is performed or not (Frank, 2006). One of the proposed functional relevancies of the hyperdirect pathway is to slow down the initial actions in cognitive control situations, when it is crucial to quickly evaluate the expected outcome of different behavioral alternatives (Frank, 2006). It is possible that the observed behavioral findings in the present study are because of the notion that the trough-coupled tACS condition indirectly interfered with the neural oscillation in the hyperdirect pathway.

At the same time, the trough-coupled tACS condition did not impair the average accuracy, but it may even have slightly improved the learning rate in one of the congruent trials, i.e., “NoGo to avoid.” We note, however, that the statistical analysis provided only inconclusive evidence for the improvement effect in the learning rate. We therefore interpret this finding that the trough-coupled tACS condition had only negligible effect if any on the “NoGo to Avoid” decisions and that the main effect of the trough-coupled tACS condition was interferential in nature.

Unexpectedly, the amplitude-modulated tACS condition slowed the learning rate for the conflicting trials, which is reminiscent of the behavioral effects of the trough-coupled tACS condition. However, its diminishing behavioral effect was less pronounced when compared with the trough-coupled tACS condition. In the amplitude-modulated tACS protocols, the slow, i.e., the θ frequency, might have played an important role in producing the cognitive effects of tACS (Minami and Amano, 2017). As increased power of θ-range oscillations leads to better performance during cognitive conflict (Cavanagh et al., 2013), we would expect behavioral improvement under this protocol. Previous studies with single-frequency θ tACS showed beneficial behavioral effects in cognitive control tasks, including reduced reaction time or facilitated behavioral accuracy (Hsu et al., 2017; Lehr et al., 2019).

Contrary to our expectations, we found no clear and consistent behavioral effects for the peak-coupled tACS protocol. In a previous study, Alekseichuk et al. (2016) observed behavioral improvement in the sensitivity index of a spatial working memory task during the peak-coupled tACS. Since the peak-coupled tACS protocol mimics the phase specificity of θ-γ CFC when signaling the need for cognitive control (Smith et al., 2015), we expected that it would increase the efficacy of the cingulate cortex to signal the need for cognitive control and thereby increase the degree of model based control implemented by the prefrontal cortex.

The lack of the behavioral effects could also have been because of the thorough instructional procedure we used in the present study. The exhaustive instructional procedure might have produced a ceiling effect, which could diminish the ability of the stimulation to further improve the performance of our volunteers. We expect that the peak-coupled tACS condition may improve the behavioral performance in groups of participants who do not reach the ceiling effect, e.g., in elderly participants or in individuals with mild cognitive impairment.

One of the limitations of the present study is that the computational modeling results were inconclusive given that the model was unable to capture our participants’ behavior. Therefore, we can neither confirm nor falsify our third hypothesis concerning the underlying cognitive processes (i.e., Pavlovian bias parameter). We speculate that the lack of fit of our computational models could be, at least partially, because of the instructional procedure we used in this study. Specifically, our participants received very thorough instructions about the task including reading the written instruction, listening to the verbal explanation of the experimenter, performing the short practice, filling out the questionnaire about the task, and performing the training session. By this procedure, we initially intended to minimize the probability that the participants would misunderstand the task and make their decisions in a random fashion. However, the exhaustive instructional procedure likely affected the strategy of the participants, who performed very well on the task. In fact, although our task was more difficult than that used in previous studies (Cavanagh et al., 2013), the overall accuracy level in the tACS sessions was higher in our study indicating that the participants were potentially able to exploit the task structure to improve their reward rate.

Evidence exists that the task instruction can indirectly influence how humans perform an instrumental learning task. This phenomenon is known in the literature as the behavioral rule-governing effect (Doll et al., 2009). It is possible that after the instructional phase at least some participants were able to infer the correct structure of the task, even before the direct experience. This may have facilitated the learning process through the mechanism of confirmation bias (Doll et al., 2009); participants learned quickly to amplify those outcomes that were consistent with their internal model of the task and discarded the incompatible ones. Given the relatively difficult reward contingency probabilities (0.65 vs 0.35), we expected much more exploration in the initial phase of the task (Csifcsák et al., 2020).

This argument is further supported by the results of the qualitative analysis we performed about the explicit knowledge of the card types. We found that all participants were able to correctly identify both the valence and the action value of the cards in the overwhelming majority of the cases (∼91%). Occasionally, the participants made mistakes when identifying the correct action to the valence (∼8%). Other error types were very rare. We interpret these findings as a further indirect support that the participants had explicit, rule-based knowledge about the structure of the task.

By using a less thorough instructional procedure, future studies may use computational modeling (Csifcsák et al., 2020) to explore the hidden parameters that may be influenced by the CFC-tACS protocol. Because these models assume that participants do gradually learn the expected value of the stimulus (Cavanagh et al., 2013; Csifcsák et al., 2020), we were not able to use them fruitfully in the present study.

Another possible limitation of the present study is the lack of a sham tACS protocol. Because real tACS can induce both cutaneous and visual perceptual adverse effects during the entire stimulation period, we preferred using a control tACS protocol, instead of a sham tACS protocol (Turi et al., 2013). The conventionally used fade-in, short-stimulation, fade-out sham protocols, may not be able to maintain effective blinding for the real intervention because of their shortness, as has been shown for transcranial direct current stimulation (Greinacher et al., 2019; Turi et al., 2019).

According to an alternative explanation, the control condition might have improved the behavioral performance to a similar extent to the peak-coupled tACS condition but slightly stronger than in the amplitude-modulated tACS condition. Given that the θ and γ tACS were continuously superimposed in the control condition, this protocol had equal chance to improve or impair the behavioral performance. Therefore, this alternative explanation does not explain why the control stimulation would have improved, rather than impaired the performance. Second, a previous study applying a closely matched control protocol found no cognitive effect on a cued-recall task, even when comparing the cognitive performance before and after the intervention (Amador de Lara et al., 2018). Therefore, we find this alternative explanation to be less likely.

Taken together, CFC-tACS protocols can extend single-frequency tACS protocols by enabling the testing of CFC phenomena intrinsic to endogenous network oscillations (Alekseichuk et al., 2016; Bächinger et al., 2017; Minami and Amano, 2017). In this study, we showed that trough-coupled tACS, i.e., when γ tACS was coupled over the trough of θ tACS, and amplitude-modulated tACS decreased the behavioral performance and the use of cognitive control in healthy participants. These findings suggest that the phase of coupling between θ and γ frequencies may play an important role in cognitive control.

10.1523/ENEURO.0126-20.2020.ed1 Extended Data 1 Extended data 1 contains all materials, pseudonymized raw data and analysis scripts used in this study that are freely available at our repository. Download Extended Data 1, ZIP file.

## Synthesis

Reviewing Editor: Macià Buades-Rotger, University of Luebeck Decisions are customarily a result of the Reviewing Editor and the peer reviewers coming together and discussing their recommendations until a consensus is reached. When revisions are invited, a fact-based synthesis statement explaining their decision and outlining what is needed to prepare a revision will be listed below. The following reviewer(s) agreed to reveal their identity: Maria Stavrinou, Séamas Weech, Nathaniel Haines.

The authors designed a study to determine how cognitive control could overcome Pavlovian bias-the automatic, reflexive tendency to approach appetitive/rewarding and avoid aversive/punishing stimuli. To do so, they had subjects undergo a probabilistic Go/No-go monetary task including both rewards and punishments, during which they used theta-gamma cross-frequency coupling via transcranial alternating current stimulation (CFC-tACS) to target brain regions previously associated with cognitive control. The authors CFC-tACS procedure utilized one of three protocols: over the peak, over the trough, and amplitude modulated tACS. They hypothesized that behavioral performance would be increased in the peak and amplitude conditions and impaired in the trough condition. Note that CFC-tACS was applied only during an initial learning phase of the task, and not in a transfer phase.

To test their hypotheses, the authors recruited 24 subjects, each of which underwent all of the task conditions described above, in addition to a fourth control condition (no CFC-tACS). Subjects and experimenters were blinded to the conditions. To analyze the task data, the authors used both hierarchical logistic regression and previously developed computational models of this particular task. They found that “the computational models were unable to capture participants behavior", but that a saturated logistic regression model (i.e. predicting trial-to-trial accuracy using logistic regression with predictors for all two- and three-way interactions between Card, tACS session and Trial, and a main effect of Session) fit the data well. Therefore, the authors decided against interpreting the computation models, and instead focused on the logistic regression results.

With logistic regression, they found that accuracy was higher in all tACS sessions and for all Cards (except the Go-to-win card in the trough condition). In general, the authors did not find strong evidence for many differences between the tACS conditions. However, they did find some evidence of decreased learning for Pavlovian incongruent cards within the amplitude modulated tACS condition relative to the control condition.

This is a well-crafted and informative study. However, there are multiple concerns related to how the authors chose to model their data, which are outlined below. Also, considering the complexity of the methods and topic, the paper could generally benefit from simpler, less jargon-loaded writing in the Introduction and Discussion.

Major concerns:

1) The authors began with a discussion of Pavlovian bias and allude to computational models that can account for it. They then fit a computational model to their data, but they discard these results claiming that “the computational models were unable to capture participants behavior”. However, they do not show the results of the model fitting, so it is difficult to understand why it failed. Given that they used a popular computational model, we believe the authors should offer more information on how/why the model failed. Perhaps a graphical representation of the model predictions versus true behavior, etc. A discussion of why the model failed is important in its own right, as it could motivate future extensions.

2) Related to (1), the task that the authors used only contained 20 trials for each of the 4 task stimuli during the training phase, and 12 trials in the subsequent transfer phase. This is a very low number of trials for this particular task. For example, the study they cite (i.e., Csifcsák, Melsæter, & Mittner, 2020) that used the same computational model used upwards of 700 trials. The tACS design might have made it difficult to include many trials due to having so many conditions, but this low number of trials may play a huge role in why the computational model performed poorly, and also likely plays a large role in their (mostly) non-conclusive results. There is no issue with null results, but it is important to emphasize that such results are expected when the data are not able to inform the parameter estimates. Looking at the posterior intervals reported in the results, most of the posterior distributions are very wide, indicating a lack of information, which likely stems from the low trial counts. The authors should make this shortcoming very clear in their discussion-namely, that the parameter estimates they have are quite imprecise, a problem that can only be solved with more data. Importantly, more data could then produce a very different set of results.

3) Another major issue concerns how the authors decided to parameterize their hierarchical logistic regression models. They mention that they used the default, uniform priors from the brms package, but uniform priors on logistic regression coefficients can have quite a strong influence on the resulting estimates. For example, see: https://onlinelibrary.wiley.com/doi/10.1111/oik.05985

This is a very important point to consider, particularly because of the low trial count as noted in (2). In cases where the data are not very informative with respect to the prior, the posterior could be very influenced by the prior choice. Therefore, some justification of the prior choice is necessary in this case.

Relatedly, it would be useful for the authors to provide either a table or a graphical depiction of all the logistic regression beta weights in their full logistic regression model. While the results section contains a lot of this information, it is useful to see visualize the uncertainty of all the parameter estimates together to get a sense of what is going on in the model.

4) It should be made explicitly clear that the tACS protocol was not based on recorded oscillations but rather on the frequency of the stimulation device. This should appear in the Abstract (e.g. “the peaks or the troughs of the *delivered* theta wave”) and at the beginning of the Methods section on the stimulation protocol. It should as well be included in the discussion in comparison to closed-loop stimulation protocols.

Importantly, the authors should generally be more clear regarding the number of conditions, and more explicit when reporting the results, especially in the Abstract.

5) Introduction: Lines 32-45. The model-free and model-based approaches should be briefly described as well. The authors describe only the Pavlovian system.

6) The introduction describes the neurophysiology of cognitive control but neglects reward processing/anticipation or approach or avoidance. The latter is not the main focus and the literature is too broad to address here, but the manuscript would benefit from a brief description of how the postulated cognitive control mechanisms interact with systems involved in the neural representation of reward.

7) The trough-peak notion should be briefly explained in the Introduction and Discussion so non-expert readers intuitively know its meaning, e.g. “over the trough (when theta power is minimal)”.

8) 2.4 Behavioral paradigm: Usually the monitor characteristics should be included as well as the design, dimensions and colors used in the stimuli (cards). Also, an example of a trial should be given and the best answer to that in both the task and the forced choice task for illustration. On a related note, was there an a-priori reason why the task was designed to be more difficult than previous versions (line 165)?

9) Lines 294-295: Which questionnaires/procedures were used for example to access the level of arousal?

10) Lines 315-319: it is beyond the scope of the paper to offer a primer on Bayesian estimation, but a brief explanation of how the HDIs are derived and a reference would orient the non-expert reader.

11) Figure 2. The authors should explain what they want to show with the figure (i.e. how and where does the current assumedly extend), and state the template used.

12) On the discussion concerning effects of amplitude-modulated tACS on the learning rate (lines 515-525). It could be interpreted that the authors believe learning was slower under conflict for AM than for control because the theta frequency (more pronounced in control than AM) should result in a benefit alone. If the control case were to impart a benefit, is it an appropriate control? (This would make the absence of a sham case more of a concern.) In general the discussion could be clearer here.

13) The Discussion could benefit from explaining why congruent trial performance would be improved following trough stimulation (lines 503-507). The discussion from line 507 onwards only addresses the decreased performance that was observed. Is there evidence of a trade-off between performance on challenging trials and the learning rate in easy trials for the other stimulation protocols?

Minor concerns:

14) Significance statement, line 26-28: the sentence “Depending on the phase of coupling” is vague, please be explicit and precise when describing the results.

15) Redish 2015 is not the primary paper for goal-directed behavior. Please choose a more classic paper that explicitly defines and deals with the topic, and *provide a brief definition of what is meant by goal-directed behavior*.

16) Please flesh out the CFC acronym on first use (line 49) and in the discussion.

17) Is the sentence beginning on line 89 an additional (4th) prediction? “We expected that amplitude-modulated tACS would improve behavioral performance by entraining the ongoing theta oscillation by the envelope of the high frequency stimulation.”

18) Please clarify the sentence on lines 433-435, as it is confusing for those that are not familiar with the task: “these coefficients reflect the situation in the Training session due to the presence of higher-order while the NoGo-to-Win card was most difficult with the other two cards being situated between.”

19) The Stan code that the authors used is a modified version of the go/no-go model code from the R hBayesDM package: https://github.com/CCS-Lab/hBayesDM/blob/master/commons/stan_files/gng_m1.stan, but the authors do not cite the original source. The authors should cite the original source (i.e. the hBayesDM paper: https://www.mitpressjournals.org/doi/full/10.1162/CPSY_a_00002)

## Author Response

We thank both the Editor and the Reviewer for their helpful, clear and constructive criticism. We believe that we addressed all the concerns, including their questions about the computational modelling and statistical analyses. We are convinced that by incorporating all these changes into the manuscript, the clarity and the flow of the text improved significantly. Below you can find our point-by-point response.

Major concerns

1.

The authors began with a discussion of Pavlovian bias and allude to computational models that can account for it. They then fit a computational model to their data, but they discard these results claiming that “the computational models were unable to capture participants behavior”. However, they do not show the results of the model fitting, so it is difficult to understand why it failed. Given that they used a popular computational model, we believe the authors should offer more information on how/why the model failed. Perhaps a graphical representation of the model predictions versus true behavior, etc. A discussion of why the model failed is important in its own right, as it could motivate future extensions.

Authors:

This is a very good point what we previously addressed in the Supplemental Material attached to the submission. However, during the submission process we found out that eNeuro does not support Supplemental Material. Therefore, we left this information in the study-associated Github repository (note that the URL to the repository is removed due to the double-blind review process). The URL will be available to the readers of the final paper. Nevertheless, we will gladly share the URL with the Reviewer if the Editor permits it (even though it breaks the double-blind review process).

In this online document, we provided a figure showing a posterior predictive check, i.e., summaries over many random datasets generated using the posterior distribution. The figure is reproduced here:

Figure. Computational modeling results. Although the model converged successfully, posterior predictive checks revealed a misfit between model-prediction and observed data.

It can clearly be seen that the generated data (thick lines with HDI-bands) are incompatible with the observed data (thin lines). We were quite puzzled by these findings as (1) the HMC chains converged successfully with none of the diagnostics showing any abnormalities and (2) we have successfully fitted this model to several datasets earlier. We believe that the reason for the misfit must lie in the behavior of our subjects in response to our instructions. Our hypothesis is that instructions were too comprehensive, which may have led the subjects using a strategy based on an understanding of the task structure (rather than gradually updating their decision probabilities as modeled by the RL models). For example, a subject could have tried to “figure out", which of the needed a go- vs. a nogo-response (i.e., giving a go-response to one card would reduce the chance that another card is a go-card). Testing this hypothesis with specifically tailored computational models is certainly possible, but out of scope for the current paper. We are investigating such alternative models in a related project and we will invite other researchers to download the raw data to test additional models on it.

We feel that including the figure illustrating the model-misfit in the main paper is not appropriate as it does not help in illustrating the main argument of the manuscript and is perhaps better located online in the Supplemental Material. We are, of course, happy to include the figure in the main text and we will leave this decision to the Editor.

2.

Related to (1), the task that the authors used only contained 20 trials for each of the 4 task stimuli during the training phase, and 12 trials in the subsequent transfer phase. This is a very low number of trials for this particular task. For example, the study they cite (i.e., Csifcsák, Melsæter, & Mittner, 2020) that used the same computational model used upwards of 700 trials. The tACS design might have made it difficult to include many trials due to having so many conditions, but this low number of trials may play a huge role in why the computational model performed poorly, and also likely plays a large role in their (mostly) non-conclusive results. There is no issue with null results, but it is important to emphasize that such results are expected when the data are not able to inform the parameter estimates. Looking at the posterior intervals reported in the results, most of the posterior distributions are very wide, indicating a lack of information, which likely stems from the low trial counts. The authors should make this shortcoming very clear in their discussion-namely, that the parameter estimates they have are quite imprecise, a problem that can only be solved with more data. Importantly, more data could then produce a very different set of results.

Authors:

We agree that the description of the number of trials was not precise enough in the previous version of the manuscript. Each of the card was presented 20 times in each block (condition), resulting in a total of 20 × 4 × 5 = 400 trials. While it is true that Csifcsák, Melsæter, & Mittner (2020) used an even longer task (20 × 4 × 9 blocks), such long tasks are unusual in the literature and data on significantly lower number of trials are routinely being analyzed with this task (e.g., 4 × 40 = 160 trials in Cavanagh et al., 2013, JoN; or 4 × 60 = 240 trials in Guitarp-Masip et al., 2012). We have clarified this by being more explicit about the total number of trials in our repeated-measure experiment. In summary, we believe that the number of trials in our experiment was adequate for estimating the coefficients and effects of interest.

The corresponding paragraph now reads (line 179-183): “Each card was presented 20 times in a random order. Independent sets of five cards were used and randomly chosen for each session from a pool of six sets of cards. We created six card sets for the scenario that one session has to be repeated. Therefore, participants performed 80 trials in each session (20 trials × four cards) and 400 trials in total (80 trials × five sessions).”

3.

Another major issue concerns how the authors decided to parameterize their hierarchical logistic regression models. They mention that they used the default, uniform priors from the brms package, but uniform priors on logistic regression coefficients can have quite a strong influence on the resulting estimates. For example, see: https://onlinelibrary.wiley.com/doi/10.1111/oik.05985

This is a very important point to consider, particularly because of the low trial count as noted in (2). In cases where the data are not very informative with respect to the prior, the posterior could be very influenced by the prior choice. Therefore, some justification of the prior choice is necessary in this case.

Relatedly, it would be useful for the authors to provide either a table or a graphical depiction of all the logistic regression beta weights in their full logistic regression model. While the results section contains a lot of this information, it is useful to see visualize the uncertainty of all the parameter estimates together to get a sense of what is going on in the model.

Authors

These are excellent points on which we are happy to elaborate. We agree that uniform priors can result in misleading inferences when data is sparse.

As we described above, our data is far from sparse and a significant number of trials is available for each subject. This is the main reason why we were not overly concerned with the choice of prior for these analyses: With enough data, reasonably chosen priors do not have a strong influence. To convince the Reviewer of that point, we have re-estimated the reported model using a) unit-information priors (i.e., standard-normal priors) on all fixed-effects and b) Cauchy (0,1) priors. Both choices are commonly recommended in the literature. The resulting coefficient-estimates are displayed in the figure below. While some estimates are slightly shrunk towards zero compared to the uniform priors, there does not appear to be a qualitative difference between these models.

Initially, we chose to report the results of the uniform prior because it is our experience that many researchers that are less experienced in Bayesian statistics prefer them compared to “subjectively chosen” priors as it can be seen as being more “objective”. We leave it up to the Editor to decide whether we should change the presentation of the results to use either of the two other priors.

Regarding the presentation of results in form of a table, we are happy to provide such a table. We are, however, uncertain how much this adds to our work as most of this information is already presented in-text. In addition, it is hard to interpret the raw regression-coefficients directly, due to the higher-order interactions present in the model. We leave the decision to the Editor as to whether the full table should be reproduced in the paper itself.

4.

It should be made explicitly clear that the tACS protocol was not based on recorded oscillations but rather on the frequency of the stimulation device. This should appear in the Abstract (e.g., “the peaks or the troughs of the *delivered* theta wave”) and at the beginning of the Methods section on the stimulation protocol. It should as well be included in the discussion in comparison to closed-loop stimulation protocols.

Importantly, the authors should generally be more clear regarding the number of conditions, and more explicit when reporting the results, especially in the Abstract.

Authors:

In agreement with the Reviewer’s suggestion, we improved the clarity of text by specifying that in the context of the present study the notion of cross-frequency coupling refers to the delivered tACS frequencies.

We incorporated these changes in the Introduction (line 71-74): “In the context of the present study, the notion of peak and trough refers to the local maximum and minimum of the amplitude of the delivered theta tACS wave, to which the short gamma tACS burst was coupled.”

5.

Introduction: Lines 32-45. The model-free and model-based approaches should be briefly described as well. The authors describe only the Pavlovian system.

Authors:

We added a brief (due to the word limit) description of the model-free and model-based approaches to the Introduction (line 41-46):

"The model-free system gradually incorporates the behavioral consequences of actions by computing the difference between the predicted and received outcome. The model-based system creates an internal world model, which enables flexible, prospective planning. Therefore, decisions do not exclusively rely on the outcome history (Helfrich & Knight, 2019).”

6.

The introduction describes the neurophysiology of cognitive control but neglects reward processing/anticipation or approach or avoidance. The latter is not the main focus and the literature is too broad to address here, but the manuscript would benefit from a brief description of how the postulated cognitive control mechanisms interact with systems involved in the neural representation of reward.

Authors:

As the Reviewer suggested, we added a brief description of this topic to the manuscript. Due to the word limit, we integrated this paragraph into the Discussion (line 552-563): “The observed behavioral effects in the present study may be due to the direct stimulation of the frontal and cingulate cortices or to indirect network effects. It has been shown in primates that there are monosynaptic connections between the frontal cortex, including the ventromedial prefrontal and cingulate cortices, to the subthalamic nucleus (Haynes & Haber, 2013). This pathway is called the hyperdirect pathway, which supposedly exert a strong top-down control on ongoing decisions: It influences whether an action is performed or not (Frank, 2006). One of the proposed functional relevancies of the hyperdirect pathway is to slow down actions in cognitive control situations, when it is crucial to quickly evaluate the expected outcome of different behavioral alternatives (Frank, 2006). It is possible that the observed behavioral findings in the present study are due to the notion that the trough-coupled tACS condition indirectly interfered with the neural oscillation in the hyperdirect pathway.”

7.

The trough-peak notion should be briefly explained in the Introduction and Discussion so non-expert readers intuitively know its meaning, e.g., “over the trough (when theta power is minimal)”.

Authors:

Thank you for the suggestion. We improved the clarity of describing peak and trough in the entire manuscript. Please see our previous response at point 4.

8.

2.4 Behavioral paradigm: Usually the monitor characteristics should be included as well as the design, dimensions and colors used in the stimuli (cards). Also, an example of a trial should be given and the best answer to that in both the task and the forced choice task for illustration. On a related note, was there an a-priori reason why the task was designed to be more difficult than previous versions (line 165)?

Authors:

We added the requested details to the manuscript (line 184-211): “The presentation of the stimuli was performed in full screen mode. We set the background color of the screen to white. At the beginning of each trial, a black fixation cross (10 or 11 s) was presented (see Figure 1A, Trial flow). Note that we used a relatively long duration of fixation cross in the present study compared with previous studies (e.g., Guitart-Masip et al., 2012). Also, during this time the participants were instructed to blink and swallow. This was a necessary step to increase the comparability of the present results with our other experiments using pre-stimulus intermittent tACS (manuscript in preparation) and scalp electroencephalogram recordings.

Then a card cue (1 s; original image size 199 × 279 pixels, presentation size 0.3 × 0.5) was presented to the participants. We used white cards and distinguished them with a black capital letter (B, C, D, F, G, H, J, K, R, S, T, V, A, E, O, U, L, M, P, Q, W, X, Y, Z) printed in the middle of the card (see Figure 1A, Trial flow). We decorated the cards by adding four pieces of simple shapes around the letter. We used rhombus, circle and rectangle shapes and filled them with blue, gray, green, pink, orange or yellow colors. In each set, we used the same shape and color for each card.

The target detection stimulus (black circle; original image size 225 × 220 pixels, presentation size 0.35 × 0.45) was shown until a response occurred, or 1 s passed. The target detection stimulus indicated to the participants that they could take the card (Go) or not (NoGo), upon which the monetary outcome depended. The feedback was displayed (original image size 402 × 205 pixels, presentation size 0.6 × 0.4) for 2 s: A green ‘+€’ sign indicated a monetary reward, a red ‘-€’ symbol indicated a monetary loss and a black horizontal bar indicated neutral monetary outcome (neither win nor loss). The next trial started 0.5 s after feedback.

In the subsequent transfer phase of the task (Figure 1B), the participants performed a two-alternative, forced-choice (2AFC) task where each card from the learning phase was paired with one of the three other cards following the order (e.g., ‘Go to win’ vs. ‘NoGo to avoid losing’, ‘NoGo to avoid losing’ vs. ‘Go to win’, etc.). Each of the 12 card pairs was presented four times until a response occurred, or 3 s passed.”

The reason for making the reward contingencies more difficult relative to earlier versions of the task was to avoid a possible ceiling effect in the behavioral performance. Given that we used a thorough instructional procedure, we expected a better performance in our experiment, compared to earlier studies. This could impede detecting behavioral improvements, supposedly induced by the peak-coupled tACS protocol.

9.

Lines 294-295: Which questionnaires/procedures were used for example to access the level of arousal?

Authors:

Thank you for the suggestions. We added the exact question and the response options to the Methods (line 290-295): “Further, we assessed the level of arousal (“How are you feeling right now?”) with a 10-point Likert-scale where value 1 corresponded to very tired and 10 to completely awake. We also assessed the presence and intensity of headache (“Do you have a headache right now?”) with an initial yes-no answer and an optional 10-point Likert-scale for yes responses. Here, value 1 corresponded to low and 10 to very strong headache.”

Also, note that in the study-related repository, we uploaded all the questionnaires.

10.

Lines 315-319: it is beyond the scope of the paper to offer a primer on Bayesian estimation, but a brief explanation of how the HDIs are derived and a reference would orient the non-expert reader.

Authors:

We agree with the Reviewer’s opinion on this issue. Therefore, we modified the paragraph in the Methods by adding a brief explanation about how we derived the HDIs and how to interpret the values (line 345-353):

"We applied Bayesian methods, and we report our results in terms of the mean of the posterior distribution and their associated 95% highest-density intervals (HDIs). These intervals are derived from the posterior distribution of the model-parameters or a combination of parameters (e.g., differences) by finding the interval that contains 95% of the posterior mass while also satisfying the criterion that all points within the interval have a higher probability density than points outside the interval (Kruschke, 2014). The interpretation of the Bayesian 95% HDI is that it gives the range in which the estimated parameter is located with a probability of 0.95. We consider effects to be statistically reliable, if the 95% HDI excludes zero.”

11.

Figure 2. The authors should explain what they want to show with the figure (i.e. how and where does the current assumedly extend), and state the template used.

Authors:

The goal of this figure was to provide a visual description of the used electrode montage, the waveforms and the produced electric field distribution in the gray matter.

In the new version of the manuscript, we linked the description with Figure 2. more explicitly in the Methods (line 272-276): “In order to estimate the magnitude of the induced electric field in the brain, we ran simulations using the free software package Simulations for Non-invasive Brain Stimulation (SimNIBS; version 3.0.2) (Thielscher et al., 2015). To this aim, we conducted electric field calculations on an anatomically realistic, six-compartment template head model (almi5.msh) available in SimNIBS.”

We also modified the legend for Figure 2 (line 282-286): “Stimulation parameters including electrode montage (A), cross-frequency-coupling tACS waveforms (B) and estimated electric field magnitudes in the gray matter. Electric field simulations were performed with SimNIBS version 3.0.2 on a template head model. The peak absolute electric field strength reached 0.3 mV/mm in the medial frontal cortex. Abbreviations: AM - amplitude-modulated; CFC - cross-frequency coupling.”

12.

On the discussion concerning effects of amplitude-modulated tACS on the learning rate (lines 515-525). It could be interpreted that the authors believe learning was slower under conflict for AM than for control because the theta frequency (more pronounced in control than AM) should result in a benefit alone. If the control case were to impart a benefit, is it an appropriate control? (This would make the absence of a sham case more of a concern.) In general the discussion could be clearer here.

Authors:

This is an interesting alternative explanation what we discuss in the Discussion (line 643-652): “According to an alternative explanation, the control condition might have improved the behavioral performance to a similar extent to the peak-coupled tACS condition but slightly stronger than in the amplitude-modulated tACS condition. Given that the theta and gamma tACS were continuously superimposed in the control condition, this protocol had equal chance to improve or impair the behavioral performance. Therefore, this alternative explanation does not explain why the control stimulation would have improved, rather than impaired the performance. Second, a previous study applying a closely matched control protocol found no cognitive effect on a cued-recall task, even when comparing the cognitive performance before and after the intervention (Amador de Lara et al., 2017). Therefore, we find this alternative explanation to be less likely.”

Note that in all four tACS conditions, the amplitude of the theta tACS was constant. We discussed this explicitly in the Methods (line 264-271). Therefore, the interesting speculation of the Reviewer “It could be interpreted that the authors believe learning was slower under conflict for AM than for control because the theta frequency (more pronounced in control than AM) should result in a benefit alone.” cannot explain this difference.

13.

The Discussion could benefit from explaining why congruent trial performance would be improved following trough stimulation (lines 503-507). The discussion from line 507 onwards only addresses the decreased performance that was observed. Is there evidence of a trade-off between performance on challenging trials and the learning rate in easy trials for the other stimulation protocols?

Authors:

We thank the Reviewer for raising this issue and in the same time, we apologize for inaccurately describing and interpreting these findings in the original submission. We had a second look at the interpretation of these results and found out that the analysis clearly indicated only a weak, i.e., inconsistent and inconclusive, evidence for the improvement effects. Consequently, interpreting these findings as a clear improvement was an overstatement, what we toned down in the revision.

We corrected the relevant paragraph in the Discussion (line 564-570):

"At the same time, the trough-coupled tACS condition did not impair the average accuracy but it may even have slightly improved the learning rate in one of the congruent trials, i.e., ‘NoGo to Avoid’. We note, however, that the statistical analysis provided only inconclusive evidence for the improvement effect in the learning rate. We therefore interpret this finding that the trough-coupled tACS condition had only negligible effect if any on the ‘NoGo to Avoid’ decisions and that the main effect of the trough-coupled tACS condition was interferential in nature.”

Minor concerns:

14.

Significance statement, line 26-28: the sentence “Depending on the phase of coupling” is vague, please be explicit and precise when describing the results.

Authors:

Thank you for the suggestion. We agree with the Reviewer that the original sentence was vague. The modified sentence (line 27-30) now reads: “We found that when gamma tACS was coupled to the trough of theta tACS, the stimulation worsened the ability of healthy participants to employ cognitive control.”.

15.

Redish 2015 is not the primary paper for goal-directed behavior. Please choose a more classic paper that explicitly defines and deals with the topic, and *provide a brief definition of what is meant by goal-directed behavior*.

Authors

In the modified sentence, we provide a brief definition of goal-directed behavior and added a new citation. The sentence now reads (line 34-36): “In goal-directed behavior, contextual and reward-related information should be effectively linked to form action plans in order to accomplish goals and perform decisions in a flexible and prospective manner (Helfrich & Knight, 2019).”

16.

Please flesh out the CFC acronym on first use (line 49) and in the discussion.

Authors

Thank you for spotting this mistake. We added the term cross-frequency coupling and the abbreviation in the first use in the Introduction (line 56-58): “Theta-gamma, phase-amplitude cross-frequency coupling (CFC) is one form of such interaction, where the phase of the theta oscillation modulates the amplitude of the gamma oscillation (Canolty & Knight, 2010).”.

17.

Is the sentence beginning on line 89 an additional (4th) prediction? “We expected that amplitude-modulated tACS would improve behavioral performance by entraining the ongoing theta oscillation by the envelope of the high frequency stimulation.”

Authors

Yes, this was our fourth hypothesis, therefore, we changed the sentence in the Introduction to make it more explicit (line 92-94): “Fourth, we expected that amplitude-modulated tACS...[].”

18.

Please clarify the sentence on lines 433-435, as it is confusing for those that are not familiar with the task: “these coefficients reflect the situation in the Training session due to the presence of higher-order while the NoGo-to-Win card was most difficult with the other two cards being situated between.”

Authors:

This sentence was the unfortunate result of a copy-and-paste error that went unnoticed, thanks for spotting it! The sentence was supposed to read: “As expected, responses to the Go-to-Win card were generally most accurate (b_GoAvo=-.88[-1.24,-0.54], b_NoGoAvo=-1.02[-1.39,-0.68], b_NoGoWin=-1.25[-1.63,-0.90]; these coefficients reflect the situation in the Training session due to the presence of higher-order interactions) while the NoGo-to-Win card was most difficult with the other two cards being situated between.”

We agree that this sentence is a bit hard to read and we therefore removed the sub-clause in the parentheses (line 466-469):

"As expected, responses to the Go-to-Win card were generally most accurate (b_GoAvo=-.88[-1.24,-0.54], b_NoGoAvo=-1.02[-1.39,-0.68], b_NoGoWin=-1.25[-1.63,-0.90]) while the NoGo-to-Win card was most difficult with the other two cards being situated between.”

19.

The Stan code that the authors used is a modified version of the go/no-go model code from the R hBayesDM package: https://github.com/CCS-Lab/hBayesDM/blob/master/commons/stan_files/gng_m1.stan, but the authors do not cite the original source. The authors should cite the original source (i.e. the hBayesDM paper: https://www.mitpressjournals.org/doi/full/10.1162/CPSY_a_00002)

Authors:

Thank you very much for pointing this out! We did indeed miss the opportunity to cite that excellent toolbox, which has facilitated much of our research! We have now added the citation to the Methods (line 383-384): “The model-code was based on a the hBayesDM toolbox (Ahn et al., 2017).”
