# Supplementary material for: θ-γ Cross-Frequency Transcranial Alternating Current Stimulation over the Trough Impairs Cognitive Control
Source: eNeuro. 2020 Sep 4;7(5):ENEURO.0126-20.2020. doi: 10.1523/ENEURO.0126-20.2020 (PMC7540931; doi:10.1523/ENEURO.0126-20.2020)
Supplement: Extended Data 1 — contains all materials, pseudonymized raw data and analysis scripts used in this study that are freely available at our repository. Download Extended Data 1, ZIP file. [file enu-eN-NWR-0126-20-s02.zip › 2020_cfc_tacs-master/experimental_materials/instructions/instruction_task_eng.docx]

**Briefing**

1. *The Card Game*

You will be presented one of 4 cards on the screen for about 1 second, followed by a black circle. Once you see the black circle, you have two answer options for each card: you can decide whether you want to take the card. To take the card, you must press the button. If you do not want to take the card, you do not have to react at all (Figure 1). One answer option for a card is 'correct', the other option is 'wrong'.

| Press the button  | Do not press the button.  |
| --- | --- |

Figure 1.

2. *Which is the right answer?*

However, you don't know at the beginning which answer option for a card is wrong or right! There is also no EXCLUSIVELY correct answer for a card, only probabilities for a correct answer option. Your task is to try and find out which of the two response options is more likely to be correct. Try to choose the answer where you think a correct answer is most likely.

3. *The feedback*

After each decision during the appearance of the circle you will receive a feedback. The green feedback stands for a performance-related remuneration of 1 point, while the red feedback results in a loss of 1 point. The grey horizontal bar means neither remuneration nor loss (Figure 2).

|  |  |  |
| --- | --- | --- |
| lose money | nothing | get money |

Figure 2.

4. *The cards predict either money gain or money loss*

Two cards give you the opportunity to win money (i.e. winning cards). On the other two cards, you can lose money (i.e. lose cards).

1. *winning tickets*

For the cards that predict that you can win money, either a green + € or a grey horizontal bar will appear as feedback. So with these cards you can either win money (indicated by the orange frame) or you don't get any additional reward (Figure 3).

|  |  |
| --- | --- |

Figure 3.

2. *lost cards*

For those cards that predict that you may lose money, either a red - € or a grey horizontal bar will appear as feedback. In other words, with these cards you can either lose money or you can avoid a loss (indicated by the orange frame, Figure 4).

|  |  |
| --- | --- |

Figure 4.

5. *The optimal strategy*

You want to select the green + € feedback for the winning cards and the grey bar for the losing cards to maximize your rewards and minimize your losses (Figure 3 and 4). You can find out by trying it out which answer option has a higher probability of winning more money or avoiding losses. Depending on the card presented, there is an optimal strategy to press the button or not (Figure 5).

Please remember that there is no single correct answer for a card. In this task you can sometimes win with a "wrong" answer, and you can also lose after a correct answer. This is because each card has a certain probability for the result. However, for each card there is an answer that is more advantageous than the other.

| Advantageous  feedback  for the winning cards | |  | Advantageous  feedback  for the loser cards | |
| --- | --- | --- | --- | --- |
|  | |  |  | |
|   ? |  |  |   ? |  |
| Optimal answer | |  | Optimal answer | |

Figure 5.

This task is not easy, so you will be asked to try all options. Don't get discouraged. Give it your best, then you will learn the correct decisions.

6. *Performance-related remuneration*

Participation in this scientific study does not entail any additional costs for you. For your participation in this scientific study you will receive an allowance of 8 Euro/hour. In addition, if you participate in the entire study, you will also receive a performance-related bonus of 12 euros (if the accuracy is over 75%).

7. *Blink and swallow*

During the task you will see the instructions "Blink and swallow*".* During this time (three seconds) you can blink, swallow and move. Please try not to move, swallow or blink during the rest of the time (about eight seconds).

8. *Do you have any further questions?*

If you have any questions before you start, please feel free to ask them now. If you have no more questions, we will start with a short questionnaire and then with the exercise session, which will take about 2 minutes. The goal of this session is to make sure that you are familiar with the program during the experiment.

9. *The cards*

Next you will see the cards used in the experiment. The goal is to become familiar with the cards. You will see each of the cards twice in a row for 3 seconds. Please look at the cards carefully, but you do not need to remember any small details.

**Briefing - Test phase**

Now is the time to test what you have learned. During this phase, a pair of cards will be presented to you on the screen at the same time. You have already seen the cards during the learning phase, but you will be presented with card combinations. Choose what you personally feel is "right" based on what you have learned in previous sessions. If you're not sure which one to take, just listen to your feelings. Press the L button to select the card on the left and the R button for the right (Figure 6). Please use the left and right index finger. You will NOT receive any feedback about your choice. This task takes about 5 minutes.

Left

Right

Figure 6.
