# Supplementary material for: θ-γ Cross-Frequency Transcranial Alternating Current Stimulation over the Trough Impairs Cognitive Control
Source: eNeuro. 2020 Sep 4;7(5):ENEURO.0126-20.2020. doi: 10.1523/ENEURO.0126-20.2020 (PMC7540931; doi:10.1523/ENEURO.0126-20.2020)
Supplement: Extended Data 1 — contains all materials, pseudonymized raw data and analysis scripts used in this study that are freely available at our repository. Download Extended Data 1, ZIP file. [file enu-eN-NWR-0126-20-s02.zip › 2020_cfc_tacs-master/experimental_materials/instructions/instructionexp_eng.docx]

**Instructions I.**

Hello and welcome to this experiment of the <information will be added after the double blind review process>! This study is registered with the Ethics Committee by a positive vote under study number 20/5/15.

Before we start, we kindly ask you to turn off your mobile phone. Please note that the vibration mode of the mobile phone can also disrupt your attention. We therefore advise against this.

*Structure of the experiment*

Overall, the whole experiment consists of several phases. Pauses are always made between the individual phases in order to allow relaxation.

1. Questionnaires (lasts approx.15 minutes)
2. Preparation for stimulation (lasts approx. 20 minutes)
3. Instruction and practice phase (lasts approx. 15 minutes)
4. Task with stimulation (lasts approx. 20 minutes)
5. Break (approx. 5 minutes)
6. Task (5 minutes)
7. Questionnaires (10 minutes)

The whole experiment will take about two hours. The experiment will take place in five sessions.

*Any questions?*

Do you have any further questions? If not, we'll start preparing now.
