# Supplementary material for: θ-γ Cross-Frequency Transcranial Alternating Current Stimulation over the Trough Impairs Cognitive Control
Source: eNeuro. 2020 Sep 4;7(5):ENEURO.0126-20.2020. doi: 10.1523/ENEURO.0126-20.2020 (PMC7540931; doi:10.1523/ENEURO.0126-20.2020)
Supplement: Extended Data 1 — contains all materials, pseudonymized raw data and analysis scripts used in this study that are freely available at our repository. Download Extended Data 1, ZIP file. [file enu-eN-NWR-0126-20-s02.zip › 2020_cfc_tacs-master/experimental_materials/questionnaires/general_questionnaire_eng.docx]

**Questionnaire before the experiment.**

Instruction. Please fill in completely and legibly. Please always mark the answer with a cross.

| 1. Age: |  |
| --- | --- |
| 2. Sex: | M  F |
| 3. Date of birth (DD/MM/YYYY): |  |
| 4. How many years have you spent in school, training and university? (in years): |  |
| 5. Did you already participat in another experiment with electrical or magnetic stimulation? | ☐ YES ☐ NO |
| 6. Are you currently participating in another experiment? | ☐ YES ☐ NO |
| 7. Do you experience headaches? | ☐ YES ☐ NO  If so, how often do you experience headaches?  ☐ Every day  ☐ Several times a week  ☐ Once a week  ☐ Several times per month  ☐ Less than once a month  Please indicate how intense your headaches are!  (1 = low - 10 = unsustainable): |

| **Questionnaire for the first session.**  Instruction. Please fill in completely and legibly. Please always mark the answer with a cross. | |
| --- | --- |
| A) **Before the session.** | |
| 1. How many hours did you sleep last night? |  |
| 2. Please estimate how well you slept on a scale from 1 to 5!   (1: very bad - 5: very good) | ☐ 1 ☐2 ☐3 ☐4 ☐5 |
| 3. How many minutes did it take you to fall asleep? |  |
| 4. How many times did you wake up last night? |  |
| 5. Have you been drinking coffee today? | ☐ YES ☐ NO  If yes, please state when you had your last coffee? |
| 6. Have you taken any medication in the last 24 hours? | ☐ YES ☐ NO  If yes, please provide the names of the medications: |
| 7. Have you been drinking alcohol in the last 24 hours? | ☐ YES ☐ NO  If yes, please state how much alcohol you have drunk:  ☐ little  ☐ moderate  ☐ much  ☐ very much |
| 8. How are you feeling right now? (1 = very tired - 10 = completely awake) | ☐ 1 ☐2 ☐3 ☐4 ☐5 6 ☐7 8 9 10 |
| 9. Do you have a headache right now? | ☐ YES ☐ NO  If so, how severe is your headache?  (1 = low - 10 = very high): |
| 10. Have you noticed any other anomalies? | ☐ YES NO☐  If yes, please call the experimenter. |

B) **After the session.**

| 1. How are you feeling right now? (1 = very tired - 10 = completely awake) | ☐ 1 ☐2 ☐3 ☐4 ☐5 6 ☐7 8 9 10 |
| --- | --- |
| 2. Did you notice any headaches during the task? | ☐ YES ☐ NO  If so, how severe was your headache?  (1 = low - 10 = very high): |
| 3. Have you noticed any other anomalies? | ☐ YES ☐ NO  If yes, please call the experimenter. |

Comments (to be filled in by staff)

| **Questionnaire for meetings two to five.**  Instruction. Please fill in completely and legibly. Please always mark the answer with a cross. | |
| --- | --- |
| A) **Before the session.** | |
| 1. How many hours did you sleep last night? |  |
| 2. Please estimate how well you slept on a scale from 1 to 5!   (1: very bad - 5: very good) | ☐ 1 ☐2 ☐3 ☐4 ☐5 |
| 3. How many minutes did it take you to fall asleep? |  |
| 4. How many times did you wake up last night? |  |
| 5. Have you been drinking coffee today? | ☐ YES ☐ NO  If yes, please state when you had your last coffee? |
| 6. Have you taken any medication in the last 24 hours? | ☐ YES ☐ NO  If yes, please provide the names of the medications: |
| 7. Have you been drinking alcohol in the last 24 hours? | ☐ YES ☐ NO  If yes, please state how much alcohol you have drunk:  ☐ little  ☐ moderate  ☐ much  ☐ very much |
| 8. How are you feeling right now? (1 = very tired - 10 = completely awake) | ☐ 1 ☐2 ☐3 ☐4 ☐5 6 ☐7 8 9 10 |
| 9. Do you have a headache right now? | ☐ YES ☐ NO  If so, how severe is your headache?  (1 = low - 10 = very high): |
| 10. Have you noticed any other anomalies? | ☐ YES ☐ NO  If yes, please call the experimenter. |

| B) **After the session.** | |
| --- | --- |
| 1. How are you feeling right now? (1 = very tired - 10 = completely awake) | ☐ 1 ☐2 ☐3 ☐4 ☐5 6 ☐7 8 9 10 |
| 2. Did you notice any headaches during the task? | ☐ YES ☐ NO  If so, how severe was your headache?  (1 = low - 10 = very high): |
| 3. Did your scalp tingling under the electrodes during stimulation? | ☐ YES ☐ NO  - If so, how much did you feel the tingling?  1 = low - 10 = very strong: |
| 4. Did your scalp itch under the electrodes during stimulation? | ☐ YES ☐ NO  If so, how badly did you feel the itching?  1 = low - 10 = very strong: |
| 5. Did your scalp burn under the electrodes during stimulation? | ☐ YES ☐ NO  If so, how much did you feel the burning?  1 = low - 10 = very strong: |
| 6. Did you notice any light flickering during the task? | ☐ YES ☐ NO  If so, how strong was this perception?  1 = low - 10 = very strong: |
| 7. Have you noticed any other anomalies? | ☐ YES ☐ NO  If yes, please call the experimenter. |

Comments (to be filled in by staff)
