# Supplementary material for: θ-γ Cross-Frequency Transcranial Alternating Current Stimulation over the Trough Impairs Cognitive Control
Source: eNeuro. 2020 Sep 4;7(5):ENEURO.0126-20.2020. doi: 10.1523/ENEURO.0126-20.2020 (PMC7540931; doi:10.1523/ENEURO.0126-20.2020)
Supplement: Extended Data 1 — contains all materials, pseudonymized raw data and analysis scripts used in this study that are freely available at our repository. Download Extended Data 1, ZIP file. [file enu-eN-NWR-0126-20-s02.zip › 2020_cfc_tacs-master/experimental_materials/questionnaires/handedness_questionnaire_end.docx]

**Edinburgh Handedness Inventory (Oldfield 1971)**

| **Participant‘s code:** |  | **Date:** |  |
| --- | --- | --- | --- |

Please indicate your preferences in the use of hands in the following activities *by putting + in the appropriate column*. Where the preference is so strong that you would never try to use the other hand unless absolutely forced to, *put ++*. If in any case you are really indifferent *put + in both columns.*

Some of the activities require both hands. In these cases the part of the task, or object, for which hand preference is wanted is indicates in brackets.

Please try to answer all the questions, and only leave a blank if you have no experience at all of the object or task.

|  | **Left** | **Right** |
| --- | --- | --- |
| **Writing** |  |  |
| **Drawing** |  |  |
| **Throwing** |  |  |
| **Scissors** |  |  |
| **Toothbrush** |  |  |
| **Knife (without fork)** |  |  |
| **Spoon** |  |  |
| **Broom (upper hand)** |  |  |
| **Stricking Match (match)** |  |  |
| **Opening box (lid)** |  |  |
|  |  |  |
|  |  |  |

**Evaluation (please leave blanked)**

| **Sum** |  |  |
| --- | --- | --- |

| **Laterality Quotient (L.Q.) L.Q .= (∑R-∑L)/( ∑R+∑L) x 100** |  |
| --- | --- |
