# Supplementary material for: θ-γ Cross-Frequency Transcranial Alternating Current Stimulation over the Trough Impairs Cognitive Control
Source: eNeuro. 2020 Sep 4;7(5):ENEURO.0126-20.2020. doi: 10.1523/ENEURO.0126-20.2020 (PMC7540931; doi:10.1523/ENEURO.0126-20.2020)
Supplement: Extended Data 1 — contains all materials, pseudonymized raw data and analysis scripts used in this study that are freely available at our repository. Download Extended Data 1, ZIP file. [file enu-eN-NWR-0126-20-s02.zip › 2020_cfc_tacs-master/experimental_materials/questionnaires/pretest_task_eng.docx]

Instruction. Please always mark the answer with a cross.

1. If you see this feedback option:

☐ The card says you can win money.

☐ The card says you can lose money.

2. If you see this feedback option:

☐ The card says you can win money.

☐ The card says you can lose money.

3. If you decide to press the button, which of the two statements is correct?

☐ I have to press the button when I see the map.

☐ I have to press the button when I see the circle.

4. If I choose the correct answer, I always get the correct feedback:

☐ The statement is correct.

☐ The statement is false.
