# Supplementary material for: θ-γ Cross-Frequency Transcranial Alternating Current Stimulation over the Trough Impairs Cognitive Control
Source: eNeuro. 2020 Sep 4;7(5):ENEURO.0126-20.2020. doi: 10.1523/ENEURO.0126-20.2020 (PMC7540931; doi:10.1523/ENEURO.0126-20.2020)
Supplement: Extended Data 1 — contains all materials, pseudonymized raw data and analysis scripts used in this study that are freely available at our repository. Download Extended Data 1, ZIP file. [file enu-eN-NWR-0126-20-s02.zip › 2020_cfc_tacs-master/experimental_materials/questionnaires/strategy_questionnaire_eng.docx]

**Questionnaire for the first session.**

**After the session.**

Did you use a strategy during the learning phase?

☐ YES

☐ NO

If 'YES' , please describe:

Did you use a strategy during the test phase?

☐ YES

☐ NO

If 'YES' , please describe:

**Questionnaire for the second to fifth meeting.**

**After the session.**

Did you use a strategy during the learning phase?

☐ YES, the same as in the first learning phase

☐ YES, but different from the first learning phase

☐ NO

If 'YES - but different from the first learning phase', please describe:

Did you use a strategy during the test phase?

☐ YES, the same as in the first test phase

☐ YES, but different from the first test phase

☐ NO

If 'YES - but different from the first test phase', please describe:
