# Supplementary material for: θ-γ Cross-Frequency Transcranial Alternating Current Stimulation over the Trough Impairs Cognitive Control
Source: eNeuro. 2020 Sep 4;7(5):ENEURO.0126-20.2020. doi: 10.1523/ENEURO.0126-20.2020 (PMC7540931; doi:10.1523/ENEURO.0126-20.2020)
Supplement: Extended Data 1 — contains all materials, pseudonymized raw data and analysis scripts used in this study that are freely available at our repository. Download Extended Data 1, ZIP file. [file enu-eN-NWR-0126-20-s02.zip › 2020_cfc_tacs-master/experimental_materials/randomization/stimulation_randomization.docx]

**The order of stimulation for each participant**

| **Participant ID** | **Session 1** | **Session 2** | **Session 3** | **Session 4** | **Session 5** |
| --- | --- | --- | --- | --- | --- |
| 1 | Training | Peak | Trough | AM | Control |
| 2 | Training | Peak | Trough | Control | AM |
| 3 | Training | Peak | AM | Trough | Control |
| 4 | Training | Peak | AM | Control | Trough |
| 5 | Training | Peak | Control | Trough | AM |
| 6 | Training | Peak | Control | AM | Trough |
| 7 | Training | Trough | Peak | AM | Control |
| 8 | Training | Trough | Peak | Control | AM |
| 9 | Training | Trough | AM | Peak | Control |
| 10 | Training | Trough | AM | Control | Peak |
| 11 | Training | Trough | Control | Peak | AM |
| 12 | Training | Trough | Control | AM | Peak |
| 13 | Training | AM | Peak | Trough | Control |
| 14 | Training | AM | Peak | Control | Trough |
| 15 | Training | AM | Trough | Peak | Control |
| 16 | Training | AM | Trough | Control | Peak |
| 17 | Training | AM | Control | Peak | Trough |
| 18 | Training | AM | Control | Trough | Peak |
| 19 | Training | Control | Peak | Trough | AM |
| 20 | Training | Control | Peak | AM | Trough |
| 21 | Training | Control | Trough | Peak | AM |
| 22 | Training | Control | Trough | AM | Peak |
| 23 | Training | Control | AM | Peak | Trough |
| 24 | Training | Control | AM | Trough | Peak |

**The order of participants selected in the data collection**

| **Participant ID** | **Participant order** |
| --- | --- |
| 1 | 16 |
| 2 | 4 |
| 3 | 19 |
| 4 | 20 |
| 5 | 22 |
| 6 | 1 |
| 7 | 10 |
| 8 | 11 |
| 9 | 14 |
| 10 | 2 |
| 11 | 17 |
| 12 | 6 |
| 13 | 7 |
| 14 | 8 |
| 15 | 21 |
| 16 | 23 |
| 17 | 12 |
| 18 | 18 |
| 19 | 24 |
| 20 | 13 |
| 21 | 5 |
| 22 | 15 |
| 23 | 3 |
| 24 | 9 |
